# Projected future changes in extreme climate indices affecting rice production in China using a multi-model ensemble of CMIP6 projections

**DOI:** 10.3389/fpls.2025.1595367

**Published:** 2025-07-17

**Authors:** Xinmin Chen, Dengpan Xiao, Yongqing Qi, Zexu Shi, Huizi Bai, Yang Lu, Man Zhang, Peipei Pan, Dandan Ren, Xiaomeng Yin, Renjie Li

**Affiliations:** ^1^ College of Geography Science, Hebei Normal University, Shijiazhuang, China; ^2^ Hebei Laboratory of Environmental Evolution and Ecological Construction, College of Geography Science, Hebei Normal University, Shijiazhuang, China; ^3^ Key Laboratory for Agricultural Water Resources, Center for Agricultural Resources Research, Institute of Genetics and Developmental Biology, Chinese Academy of Sciences, Shijiazhuang, China; ^4^ Hebei Key Laboratory for Agricultural Water Saving, Center for Agricultural Resources Research, Institute of Genetics and Developmental Biology, Chinese Academy of Sciences, Shijiazhuang, China; ^5^ Engineering Technology Research Center, Geographic Information Development and Application of Hebei, Institute of Geographical Sciences, Hebei Academy of Sciences, Shijiazhuang, China; ^6^ School of Resources and Environment, College of Carbon Neutrality, Linyi University, Linyi, China

**Keywords:** rice system, extreme climate indices, multi-model ensemble, global climate model, CMIP6

## Abstract

Assessing and predicting the spatial-temporal characteristics of extreme climate events can effectively identify the impacts of climate change on crop production and propose targeted measures. This study systematically evaluates the intensity and spatiotemporal evolution of extreme climate events during critical phenological stages in China’s major rice-growing regions based on 11 extreme climate indices (ECIs). The future climate data were obtained from 18 Global Climate Models (GCMs) integrated in the Coupled Model Intercomparison Project phase 6 (CMIP6) with four shared socio-economic pathways (SSPs) to project the future changes of ECIs related rice production. The results indicate that the multi-model ensemble constructed via the Independence Weighted Mean method (IWM) significantly outperformed both the arithmetic mean method (AM) and individual GCMs in replicating observed trends of 11 ECIs during the historical period (1981–2014), with notable reductions in root mean square error (RMSE) for certain indices. The projections reveal that under the SSP585 scenario, the duration of extreme heat events (e.g., HD) in southern China will increase by 12–18 days by the 2080s compared to the historical period (1981–2014), representing the highest increase among all scenarios. The extreme drought events (e.g., D-Vgp) in northeastern China are projected to reach 14.8, 9.7, and 9.7 days by the 2040s, further rising to 14.3, 10.0, and 10.3 days by the 2080s. The extreme precipitation events are predominantly concentrated in southwestern and southern China, with consecutive wet days (CWD) showing limited increase within 3 days. The findings highlight that China’s rice cultivation will face intensified extreme climate challenges in the future, particularly extreme heat stress, necessitating urgent adaptive strategies to mitigate the adverse impacts of climate change on rice production.

## 1 Introduction

The Special Report on Global Warming of 1.5°C from the Intergovernmental Panel on Climate Change’s (IPCC) Sixth Assessment Report indicates that anthropogenic climate change has already elevated global surface temperatures by approximately 1.0°C (0.8–1.2°C) above pre-industrial levels. Furthermore, the report projects that global warming is likely to reach 1.5°C between 2030 and 2052 under current emission trajectories (IPCC, 2018). Several empirical studies confirm that rising temperatures do lead to an increase in the frequency and severity of extreme climate events (Almazroui et al., 2020; Dong et al., 2018; Wu et al., 2020). Previous research has found that changes in precipitation increased linearly with the increase in temperature, and the frequency of extreme precipitation also increased significantly (Knutti et al., 2016). Specially, China has witnessed a 10% surge in extreme precipitation (extreme precipitation is determined by relative threshold method) since 2000 (Jian et al., 2021), and this increase has been particularly significant in the middle and high latitudes (Allen and Ingram, 2002; Trenberth, 2011). Agricultural production is sensitive to extreme climate events, which can significantly impact crop cultivation and jeopardizing food security (Bai et al., 2021; Xiao et al., 2022; Chen et al., 2024). Generally, the effects of extreme climate events on crop yield are stronger and more harmful than that of the average climate. Related studies analyzing historical data have indicated that extreme climate change has caused significant fluctuations in global food production on an annual basis, especially in South Asia and China (Shi et al., 2024; Donat et al., 2017; Gao et al., 2002; McPhillips et al., 2018; Orlowsky and Seneviratne, 2012).

Rice production is critical for global food security, as it serves as a staple food for more than half of the world’s population (De Vos et al., 2023). As the world’s largest producer and consumer of rice, China produced over 28% of the total global rice supply, and more than 65% of Chinese households rely on rice as their staple food (National Bureau of Statistics of China, 2017). China’s production volatility directly impacts global markets (Godfray et al., 2010). In recent decades, the frequency and intensity of extreme climate events during the growth of rice have significantly increased, and their impact on rice production in China has become increasingly obvious (Zhai et al., 2005; Xiong et al., 2016; Tao and Zhang, 2013). During the heading and flowering period of rice, the extreme high temperature may lead to a sharp decline in photosynthesis and an increase in transpiration, leading to flower and fruit abortion, resulting in a higher empty shell. In addition, low temperature damage at the filling period and maturity period may delay the maturity (Guo et al., 2019). The study have shown that rice yield has decreased by 1.5–9.7% due to heat stress over the past three decades in China (Shi et al., 2015; Zhang et al., 2016). In contrast, the average intensity of rice exposure to cold stress during the near future (2021–2050) under the Representative Concentration Pathways (RCP) 8.5 scenario is projected to be lower than that during the historical period of 1980–2008 (Wang et al., 2016). In addition, rice cultivation is highly sensitive to precipitation (Wang et al., 2018). Relevant studies indicated that the frequency of extreme precipitation decreased in the rice producing areas of northeast and southwest China from 1951 to 2004, while the frequency of extreme precipitation increased in the western region and the middle and lower reaches of the Yangtze River in South China (Jian et al., 2020). Rice-growing regions are also at risk of increasing drought trends in China (Li et al., 2009; Tao et al., 2013a). In the future, rice will face more frequent extreme temperatures and simultaneous stresses of drought and flood during its critical development stages (Rang et al., 2011). Although existing study has clarified the correlation between extreme climate events and rice production in historical periods, there is a lack of quantitative analysis on future extreme climate events during rice phenological stages and their temporal and spatial variation characteristics.

Global Climate Models (GCMs) have been widely used for climate change impact assessments (Jiang et al., 2012; Sillmann and Roeckner, 2008). However, their coarse temporal and spatial resolutions increase prediction uncertainties, particularly in China’s major rice-producing regions characterized by complex topography and intensive cultivation systems, where these limitations severely constrain agriculture impact assessments at regional scales (He et al., 2018). Therefore, it needs a method to optimize the limitations of different GCMs for assessing future climate condition (Knutti and Sedlacek, 2013; Tebaldi and Knutti, 2007). Recent studies have demonstrated that multi-model ensemble approaches can optimize the limitations of individual GCM by combining their predictive strengths through weighted methods (Asseng et al., 2013; Li et al., 2015a). Specifically, multi-model ensembles could combine results from various GCMs using weighted methods such as Bayesian methods to reduce uncertainties (Tebaldi et al., 2005). Among these, the Independent Weighted Mean method (IWM) developed by Bishop and Abramowitz (2013) has shown superior performance over traditional arithmetic averaging method by accounting for inter-model dependencies through error covariance matrices. Recent relevant empirical studies have proved that this progress is particularly valuable in the assessment of agricultural climate risks (Bai et al., 2021; Xiao et al., 2022; He et al., 2018).

Despite these methodological advancements, existing research predominantly focuses on single climatic factors, lacking comprehensive analysis of combined climate stresses (e.g., heat stress, cold damage, drought and flooding) during critical rice phenological stages, especially the quantitative and spatio-temporal evolution characteristics of extreme climate events during rice production under different emission scenarios (Zhao et al., 2022). The extreme climate index (ECI) can characterize the intensity and frequency of extreme climate events stress during rice growth period. The scientific assessment of extreme climate events during key phenological periods in major rice producing areas in China based on ECI can provide a reference for the government and farmers to rationally arrange rice production and cope with climate risks. Therefore, this study employs daily climate data from 18 GCMs provided by the Coupled Model Intercomparison Project Phase 6 (CMIP6), combined with the Independent Weighting Method (IWM) to construct 11 representative ECIs. These indices quantitatively characterize climate stress intensity and frequency across the entire rice growth, spanning historical (1981-2014) and future periods (2031-2060, 2071-2100) under four SSP scenarios. The primary objectives of this study are: (1) to predict the stress of extreme climate events (e.g., heat stress, cold stress, drought stress, and precipitation stress) defined by ECIs in the 21st century; (2) to explore projected temporal trends and spatial patterns of future ECIs under different warming scenarios. The findings will enhance understanding of climate change risks, particularly the impacts of extreme climate events on China’s rice cultivation regions, and facilitate the translation of climate projections into actionable agricultural adaptation strategies.

## 2 Materials and methods

### 2.1 Study area

**Figure 1:**
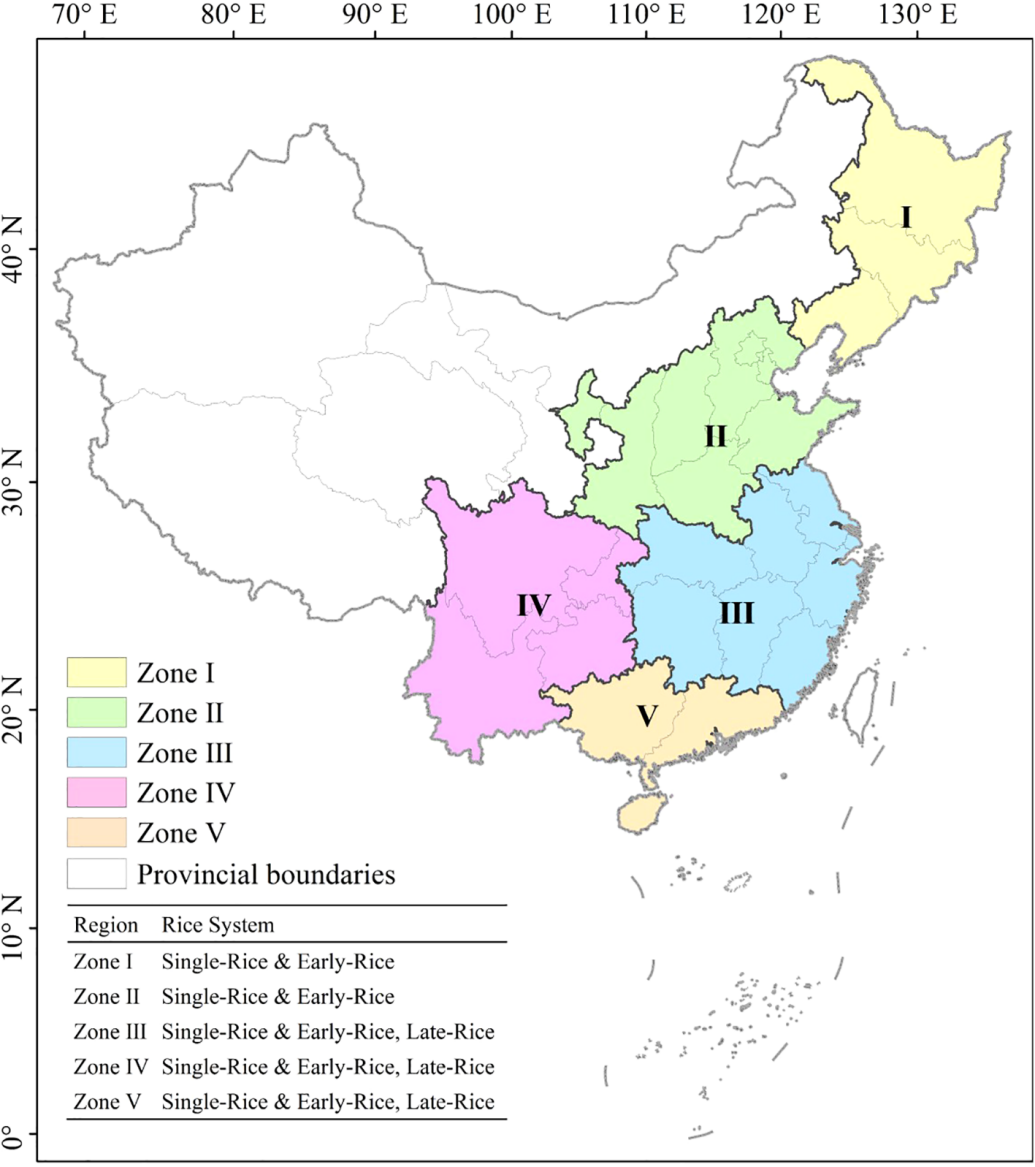
The spatial distribution region of rice cultivation in China. The specific rice system is shown in the table at the bottom left of the figure.

The study areas in China exhibit diverse environments and rice planting structures, characterized by varying cropping intensity and cultivation practices. Taking these differences into account, the entire study area was divided into five subregions according to rice cropping system and growing environment (**Supplementary Table S1**). Specifically, the early-rice and single-rice areas include Zone I, Zone II, Zone III, Zone IV and Zone V, and the late-rice areas include Zone III, Zone IV and Zone V (Luo et al., 2020). Due to the spatial overlap of the planting areas of early-rice and late-rice, in order to facilitate the display of results, we classify early-rice and single-rice into one category, and late-rice into one category. To ensure clarity in result visualization, early-rice and single-rice were amalgamated into a single legend category based on their spatial overlap characteristics (**Figure 1**).

### 2.2 Climate data

We selected 18 GCMs (**Table 1**) which provided both historical and future climate projections from CMIP6 based on the availability and completeness, focusing on daily total precipitation, daily mean temperature (Tmean) and maximum temperature (Tmax). The historical data from 1981–2014 were selected as the historical period. In order to capture representative climate scenarios in the future, four Shared Socioeconomic Pathways (SSPs), including SSP126, SSP245, SSP370 and SSP585, were selected in this study. The SSP126 represents the updated Representative Concentration Pathway 2.6 (RCP2.6) scenario, assuming that sustainable development is achieved through global cooperative governance and a radiative forced stability of 2.6 W m^-2^ is realized by 2100. The SSP245 represents the mid-path development trajectory of climate policy implementation, corresponding to the moderate socio-economic challenges and 4.5 W m^-2^ forcing of RCP4.5. The SSP370 maintains a regional competition path characterized by the adoption of lenient climate policies and the mitigation of greenhouse gas emissions, combining a strong reliance on fossil fuels with 7.0 W m^-2^ forcing in 2100. Finally, the SSP585 reflects the fossil fuel development model of RCP8.5, predicting unconstrained growth driven by high energy demand to reach 8.5 W m^-2^ in 2100, presenting the worst-case scenario without policy intervention (Eyring et al., 2016; Meinshausen et al., 2011). This scenario selection can conduct a comprehensive assessment of climate impact under different emission limits and social response frameworks. To address differences in spatial resolution among the different GCMs, we employed the Double Line Interpolation Method (DLIM) to interpolate the data onto a uniform grid of 0.25°×0.25°. Subsequently, the Delta Change Method (DCM) was applied to perform monthly deviation correction for CMIP6 data, using the historical period of 1981–2014 as the base for deviation correction.

**Table 1:** The 18 global climate models (GCMs) used in this study.

Code	GCM name	Abbreviation	Country	Spatial resolution
1	ACCESS-CM2	ACC1	Australia	1.88°×1.25°
2	ACCESS-ESM1-5	ACC2	Australia	1.88°×1.25°
3	CanESM5	CAN	Canada	2.81°×2.79°
4	CMCC-ESM2	CMC	Italy	1.25°×0.94°
5	EC-Earth3	ECE1	Europe	0.7°×0.7°
6	EC-Earth3-Veg	ECE2	Europe	0.7°×0.7°
7	EC-Earth3-Veg-LR	ECE3	Europe	1.13°×1.12°
8	FGOALS-g3	FGO	China	2.8°×2.8°
9	CFDL-ESM4	GFD	United States	1.25°×1°
10	INM-CM4-8	INM1	Russia	2°×1.5°
11	INM-CM5-0	INM2	Russia	2°×1.6°
12	IPSL-CM6A-LR	IPS	France	2.5°×1.27°
13	KACE-1-0-G	KAC	South Korea	1.88°×1.25°
14	MIROC6	MIR	Japan	1.41°×1.4°
15	MPI-ESM1-2-HR	MPI1	Germany	0.94°×0.94°
16	MPI-ESM1-2-LR	MPI2	Germany	1.88°×1.86°
17	MRI-ESM2-0	MRI	Japan	0.94°×0.94°
18	NorESM2-MM	NOR	Norway	1.25°×0.94°

To evaluate the ability of each GCM in simulating extreme climatic events in rice-growing areas of China over historical periods, daily total precipitation, daily mean temperature and maximum temperature data (https://cds.climate.copernicus.eu/) from the reanalysis dataset ECMWF Reanalysis v5 (ERA5) were used as observations. ERA5 was the latest reanalysis dataset by the European Centre for Medium-Range Weather Forecasts (ECMWF), offering a spatial resolution of 0.25°×0.25° and a temporal resolution of 1 hour. Its high resolution is particularly suitable for capturing regional climate variability in China, including the rice-growing areas. ERA5 has been extensively validated against observational data in previous studies, demonstrating its reliability for temperature, precipitation, and extreme climate event analysis in East Asia, including China (Xu et al., 2022).

### 2.3 Phenology data

In order to accurately assess extreme climate events during rice growth period, three key phenological stages of rice (i.e., transplanting date, heading date and maturity date) were combined in this study. Rice phenology data are sourced from the ChinaCropPhen1km dataset (https://figshare.com/), which is a comprehensive dataset of crop phenology dataset in China from 2000 to 2019. The dataset was developed using Global Land Surface Satellite (GLASS) Leaf Area Index (LAI) products and has been validated against agricultural meteorological stations operated by the China Meteorological Administration, demonstrating high accuracy with an error of less than 10 days (Luo et al., 2020). In this study, the multi-year average values of the three phenological stages from 2000 to 2019 were calculated, with a spatial resolution of 1km. Subsequently, the average phenological values were aggregated at the 0.25°×0.25° grid scale to obtain the rice phenological data used in this study (**Supplementary Figures S1, S2**). Due to different growth stages of rice have different sensitivity to climate conditions, we divided the whole growth stage into two stages, namely vegetative growth period from transplanting to heading, and reproductive growth period from heading to maturity.

### 2.4 Extreme climate indices

To systematically assess the frequency and intensity of extreme climate events during critical phenological phases of rice, this study selected 11 Extreme Climate Indices (ECIs) with explicit agronomic significance based on rice physiological vulnerability and disaster chain transmission mechanisms. The definitions of these indices are based on a synthesis of previous research and national climate and crop standards. Details of these 11 ECIs are shown in **Table 2**. These ECIs include 7 extreme temperature indices, i.e., hot days (HD), consecutive hot days (HCD), extreme heat days (EHD), consecutive extreme heat days (ECD), heat degree days (HDD), mild cold days (MCD) and severe cold days (SCD). The seven temperature-related indices were developed according to the national standard file and previous empirical study (GB/T 27959-2011, 2012; He et al., 2018; Huang et al., 2017). In addition, the vegetative phase drought index (D-Vgp) and the reproductive phase drought index (D-Rgp) were established following the standard of classification for drought severity (SL424-2008, 2009), and 2 extreme precipitation indices, heavy precipitation days (HPD) and consecutive wet days (CWD) from the definition and classification of rainstorm by China Meteorological Administration to evaluate waterlogging and disease risks (CMA, 2012). Based on the average phenology of rice from 2000 to 2019, we calculated these ECIs for the historical period and the future period across the five rice-growing subregions.

**Table 2:** Definitions of the 11 extreme climate indices (ECIs) used in this study.

ID	Abbreviation	Index	Definition	Units
1	HD	Hot days	The number of days with Tmean ≥ 30 °C from heading to maturity	d
2	HCD	Consecutive hot days	The number of days with three or more continuous days of Tmean ≥30 °C from heading to maturity	d
3	EHD	Extreme heat days	The number of days with Tmax ≥ 35°C from heading to maturity	d
4	ECD	Consecutive extreme heat days	The number of days with three or more continuous days of Tmax ≥ 35°C from heading to maturity	d
5	HDD	Heat degree days	The sum of the degrees by which Tmax ≥ 35°C over three or more continuous days from heading to maturity	°C
6	MCD	Mild cold days	The number of days with two or more continuous days of Tmean ≤ 17 °C from transplanting to heading	d
7	SCD	Severe cold days	The number of days with three or more continuous days of Tmean ≤ 22 °C from heading to maturity	d
8	D-Vgp	Drought events in vegetative phases	The number of days with daily precipitation<1 mm with ten or more days from transplanting to heading	d
9	D-Rgp	Drought events in reproductive phases	The number of days with daily precipitation<1 mm with ten or more days from heading to maturity	d
10	HPD	Heavy precipitation days	The number of days with daily precipitation ≥ 50 mm from transplanting to maturity	d
11	CWD	Consecutive wet days	The number of days with five or more continuous days of daily precipitation ≥ 20 mm from transplanting to maturity	d

### 2.5 Bias correction

GCMs have been used in many studies of historical climate evolution and climate change projections under future different emission scenarios (Hamadalnel et al., 2022). However, the systematic deviation of GCM will greatly affect the reliability of historical simulation and future prediction, so it is an effective means to improve the evaluation effect of regional climate change model by correcting the systematic error of GCMs and then conducting simulation and prediction. The Delta Change Method (DCM) was commonly method used to conduct bias correction (Maraun and Widmann, 2018). The formula for temperature variables as follows (Equation 1):


(1) $$T_{sim}^{DCM}(y,m,d)=T_{sim}(y,m,d)+({\bar{T}}_{obs}^{his}(m)-{\bar{T}}_{sim}^{his}(m))$$

Precipitation is bounded below by zero and covers different orders of magnitude across different regions. A multiplicative rather than additive bias correction is therefore more common when applying the DCM for precipitation, which corresponds to applying the simulated relative change to the observations (Maraun and Widmann, 2018). The formula for precipitation is estimated as (Equation 2):


(2) $$\begin{array}{c} P_{sim}^{DCM}(y,m,d)=P_{sim}(y,m,d)\times \frac{{\bar{P}}_{obs}^{his}(m)}{{\bar{P}}_{sim}^{his}(m)} \end{array}$$

where *y* (*y* = 1981, 1982… 2100) denotes the year; *m* (*m* = 1, 2…12) is the *m*th month of the *y*th year; *d* is the *d*th day of the *m*th month of the *y*th year. obs  was the observational data (i.e., ERA5 data); his  was the history data of GCMs; *sim* was the simulated data of GCMs. ${\bar{P}}_{obs}^{his}(m)$ is the multi-year monthly average precipitation of *m*th month of the observation data during the historical period (1981–2014); ${\bar{P}}_{sim}^{his}(m)$ is the multi-year monthly average precipitation of *m*th month of GCMs during the historical period (1981–2014); ${\bar{T}}_{obs}^{his}(m)$ is the multi-year monthly average daily temperature of *m*th month of the observation data during the historical period (1981–2014); ${\bar{T}}_{sim}^{his}(m)$ is the multi-year monthly average daily temperature of *m*th month of GCMs during the historical period (1981–2014). $P_{sim}^{DCM}(y,m,d)$ and $T_{sim}^{DCM}(y,m,d)$ were used for bias correction of precipitation data and daily temperature (e.g., average temperature and maximum temperature) data, respectively.

### 2.6 Multi-model ensemble method

Due to the complexity of the climate system, an individual global climate model (GCM) often fails to adequately and accurately describe climate change. The combined results from multiple models are based on the underlying assumption that errors tend to cancel out when the models are independent (Wang et al., 2016). In this study, Arithmetic Mean (AM) and Independence Weighted Mean (IWM) (Bishop and Abramowitz, 2013) are employed to generate multi-mode ensemble of ECIs. The IWM determines the optimal weighted coefficients for each GCM by minimizing the Mean Square Error (MSE) between the ensemble results and observed values. The calculation formula is as follows (Equations 3 and 4):


(3) $$\begin{array}{c} \sum_{j=1}^{J}{\left(\mu_{e}^{j}-y^{j}\right)}^{2} \end{array}\;$$

(4) $$\mu_{e}^{j}=w^{T}x^{j}=\sum_{k=1}^{K}w_{k}x_{k}^{j}$$

where *j* (*j* = 1, 2… *j*) is the time interval of ECIs, *k* (*k* = 1, 2… *k*) is the number of GCM pattern categories (in this study, j=34 and k=18); $\mu_{e}^{j}$ is the multi-model ensemble result of ECIs in the *j*th year; $y^{j}$ is the ECIs observed in the *j* ^th^ year; $w={\left[w_{1},w_{2},\;\ldots ,w_{k},\;\ldots ,w_{K}\right]}^{T}$ ; $x^{j}={\left[x_{1}^{j},x_{2}^{j},\ldots x_{k}^{j},\ldots x_{K}^{j}\right]}^{T}$ ; $w_{k}$ is the coefficient of the *k*th linear combination of GCMs; $x_{k}^{j}$ is the ECIs of year *j* in the *k* ^th^ GCM model. In addition, in order to make sure $\sum_{k=1}^{K}w_{k}=1$ , this constraint term is solved using the Lagrange multiplier (λ):


(5) $$\begin{array}{c} F(w,\lambda )=\frac{12}{\;}[\frac{1}{\text{J}-1}\sum_{j=1}^{J}{\left(\mu_{e}^{j}-y^{j}\right)}^{2}]-\lambda ((\sum_{k=1}^{K}w_{k})-1) \end{array}$$

The solution of Equation 5 can be expressed as:


(6) $$\begin{array}{c} w=\frac{A^{-1}1}{1^{T}A^{-1}1} \end{array}$$

(7) $$\begin{array}{c} A=\frac{\sum_{k=1}^{K}(x^{j}-y^{j}){\left(x^{j}-y^{j}\right)}^{T}}{J-1} \end{array}$$

where $1^{T}=[1,1,\ldots ,1]$ ; A is the sample-based estimate of the covariance of the bias-corrected errors between all the ensemble members (Equations 6 and 7).

However, due to the characteristics of HDD, certain regions may not be affected by extreme climatic events, resulting in some GCM data from CMIP6 having no value at specific grid points. Hence, when performing the multi-model ensemble using IWM, the simulation results for HDD will be missing. Therefore, we only calculate the arithmetic mean of multiple GCMs for HDD.

### 2.7 Data evaluation method

The Taylor diagram (Taylor, 2001) is a graphical representation that allows for a visual comparison the differences between model data and observed data. In this study, the observational reference data were obtained from the ERA5 reanalysis dataset provided by the European Space Agency (ESA). The ERA5 dataset serves as a benchmark for evaluating the statistical performance of model outputs, including metrics such as correlation coefficients, standard deviation, and root-mean-square error (RMSE) relative to actual observations.

In this study, the Taylor diagram is used to assess the reliability of the 11 ECIs calculated from the 18 GCMs, the multi-model ensemble results using Arithmetic Mean (AM), and Independent Weighted Mean (IWM). The correlation coefficient (R), standard deviation (SD) and root mean square error (RMSE) between the ECIs simulated by CMIP6 model and the ECIs calculated by ERA5 data were analyzed for the historical period (1981-2014). The RMSE is defined by the following formula (Equation 8):


(8) $$\begin{array}{c} RMSE=\sqrt{\frac{\sum_{j=1}^{n}{\left(X_{sim,j}-X_{obs,j}\right)}^{2}}{n}} \end{array}$$

where $X_{sim,j}$ is the simulation value of the *j*th year, $X_{obs,j}$ is the corresponding reanalysis data for the *j*th year.

**Figure 2:**
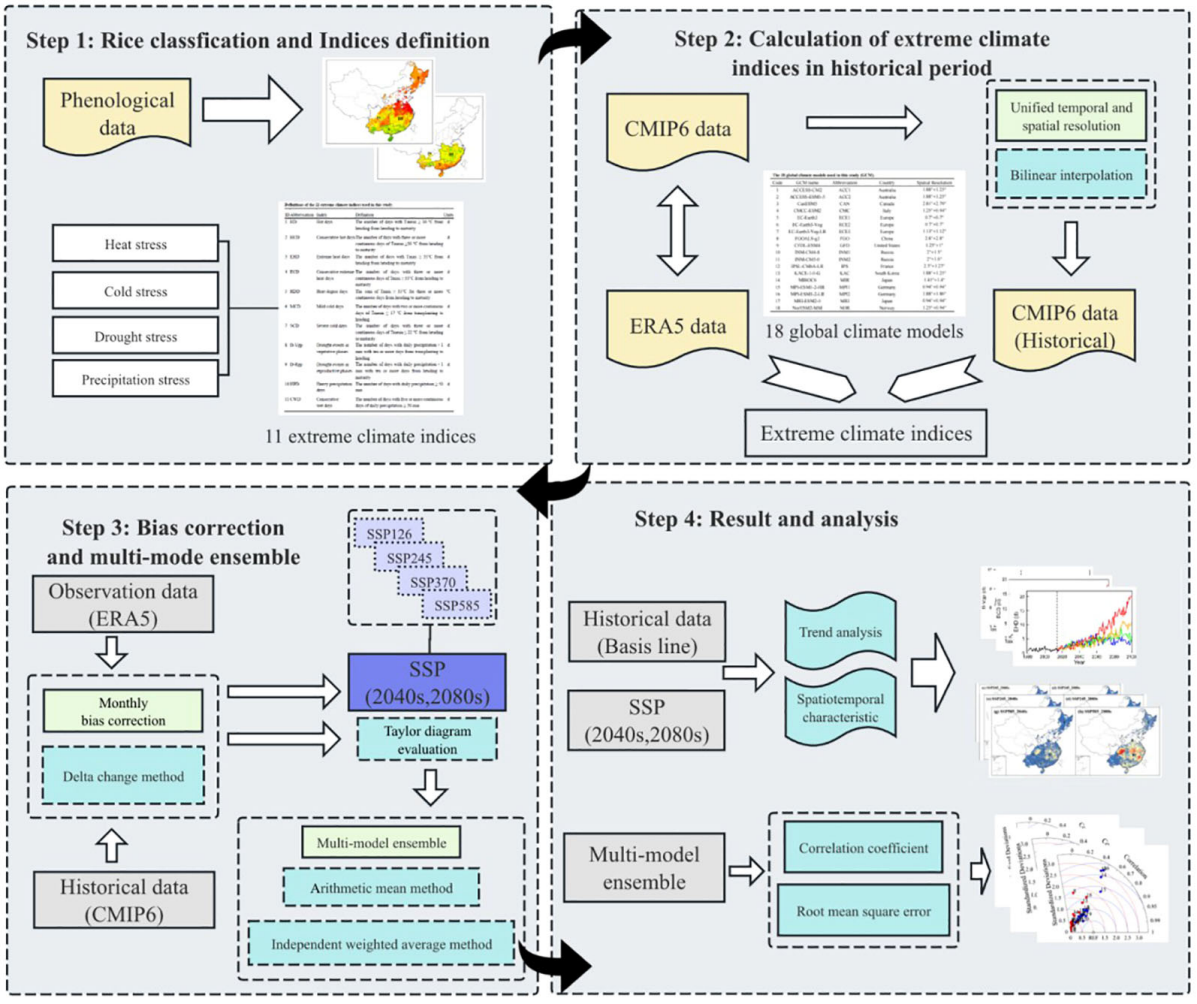
Flow chart of multi-model ensemble of CMIP6 projections for future extreme climate stress on rice under different climate scenarios in China.

Overall, **Figure 2** illustrates the flow chart to project future extreme climate stress for rice based on CMIP6 projections under different warming scenarios in China.

## 3 Results

### 3.1 Spatiotemporal variation of historical extreme climate events affecting rice production

**Figure 3:**
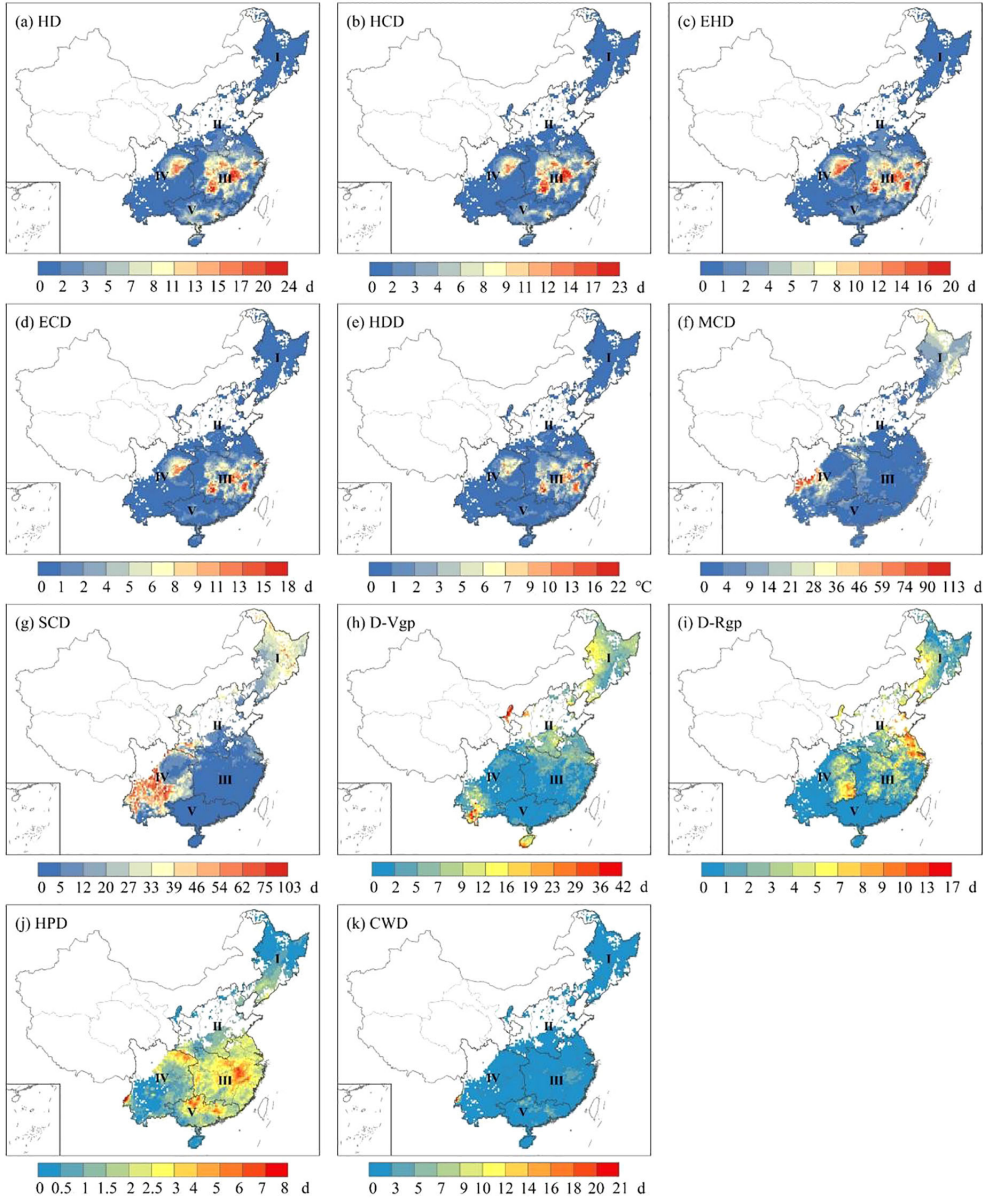
The spatial distribution of extreme heat stress **(a-e)**, extreme cold stress **(f, g)**, extreme drought stress **(h, i)** and extreme precipitation stress **(j, k)** for single-rice and early-rice during historical period.

**Figure 4:**
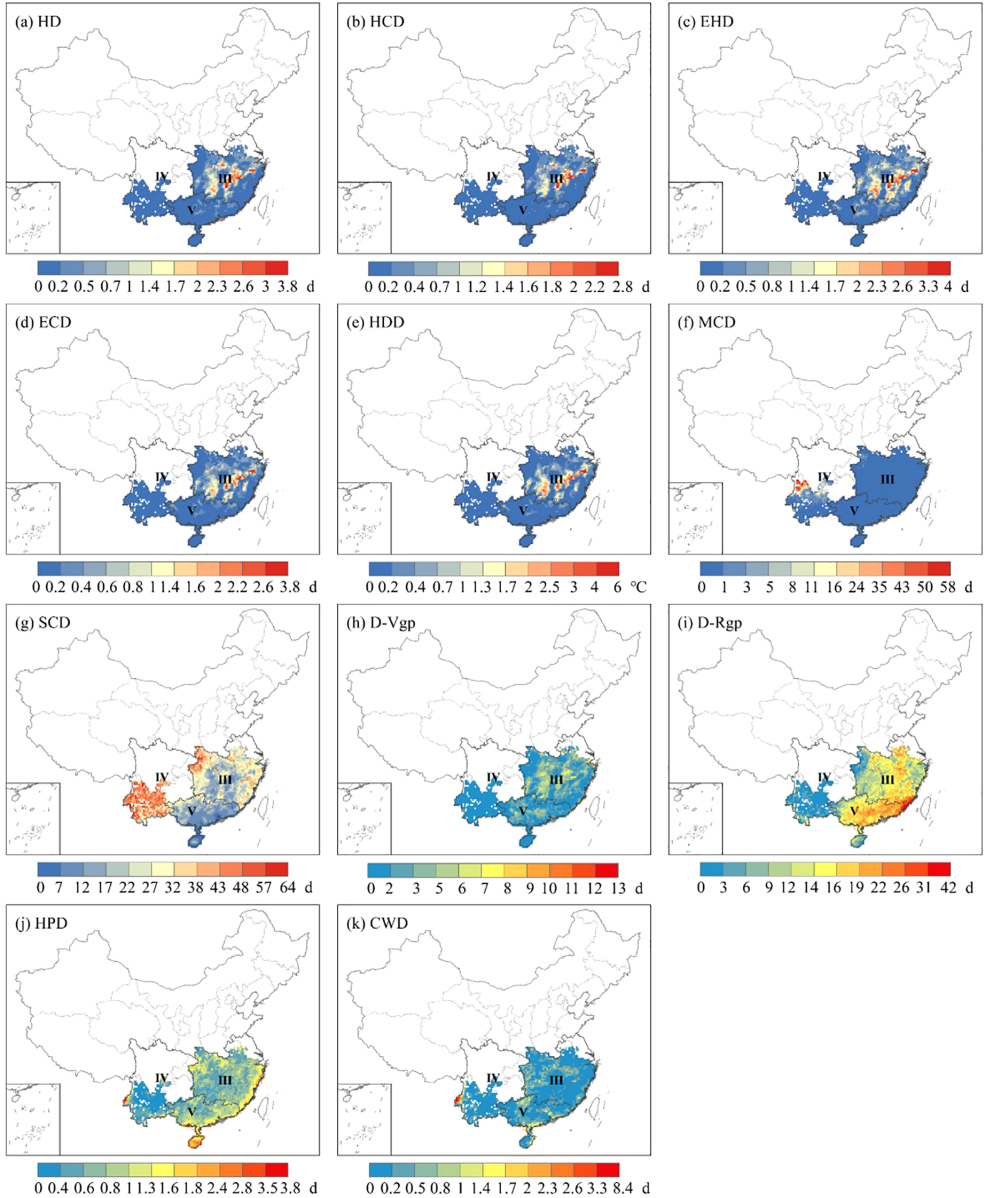
The spatial distribution of extreme heat stress **(a–e)**, extreme cold stress **(f, g)** extreme drought stress **(h, i)** and extreme precipitation stress **(j, k)** for late-rice during historical period.

During the historical period from 1981 to 2014, based on the ERA5 reanalysis dataset, extreme heat stress became the primary threat in the critical period of rice growth, and it was most significant in Zone III and Zone IV. In contrast, the duration of HD was limited to around 8 days in other regions such as Zone I, Zone II, and Zone V (**Figure 3a**). The highest temperature of HDD varied across the five zones, with values of 0.4 °C, 6.0 °C, 21.5 °C, 9.9 °C and 2.25 °C for Zone I, Zone II, Zone III, Zone IV and Zone V, respectively (**Figure 3e**). The central of Zone III, encompassing the Yangtze River basin, experienced the highest accumulated temperature, which is the major rice producing area in southern China. On the other hand, the late-rice in south China experienced less extreme heat stress than single-rice and early-rice in the growth period due to its later growing period (**Figures 4a, e**). Overall, the spatial distribution of HCD, EHD and ECD showed similar characteristics (**Figures 3b–d**, **4b–d**), while the southern rice growing area was more exposed to extreme heat stress than the northern one.

For single-rice and early-rice, cold stress was concentrated in Zone I and Zone IV (**Figures 3f, g**). The maximum duration of SCD was 62.4 days, 67.4 days, 36.2 days, 103.0 days and 1.5 days for Zone I, Zone II, Zone III, Zone IV and Zone V, respectively. As for late-rice, the maximum duration of SCD was 69.8 days, 74.2 days and 37.6 days (**Figure 4g**). The intensity and extent of cold stress were higher for SCD compared to MCD (**Figures 4g, f**).

For single-rice and early-rice, the most pronounced drought stress areas located in the western of Zone I, with other affected areas including the western of Zone II, and the southern of Zone IV and Zone V in D-Vgp (**Figure 3h**). Severe drought stress was observed in west-southern of Zone I, as well as from the eastern of Zone II to Zone III, the eastern of Zone IV also cannot be ignored drought stress either in D-Rgp (**Figure 3i**). In the case of late-rice, the duration of drought stress exceeded that of D-Vgp in terms of both spatial distribution and intensity persistence in D-Rgp (**Figures 4h, i**). The maximum values of the D-Rgp were 42.2 days, 16.0 days and 26.7 days in Zone III, Zone IV and Zone V, respectively.

Precipitation stress showed a greater variation in the five zones. For single-rice and early-rice, the southern regions, primarily Zone III, Zone IV and Zone V, faced more intense precipitation stress in terms of HPD (**Figure 3j**). In general terms, the average number of CWD across five zones was less than 1 (**Figure 3k**). As for late-rice, the prolonged duration of precipitation stress in terms of HPD was distributed along the edges of Zone III, Zone IV, Zone V (**Figure 4j**). The maximum days of precipitation stress also occurred in western of Zone IV for CWD (**Figure 4k**), but the average number days was 0.7 days, 0.3 days, 0.8 days, respectively. In summary, both the duration and intensity of precipitation stress for late-rice were greater than that for single-rice and early-rice.

### 3.2 Comparison between observed and simulated extreme climate indices in 1981–2014

**Figure 5:**
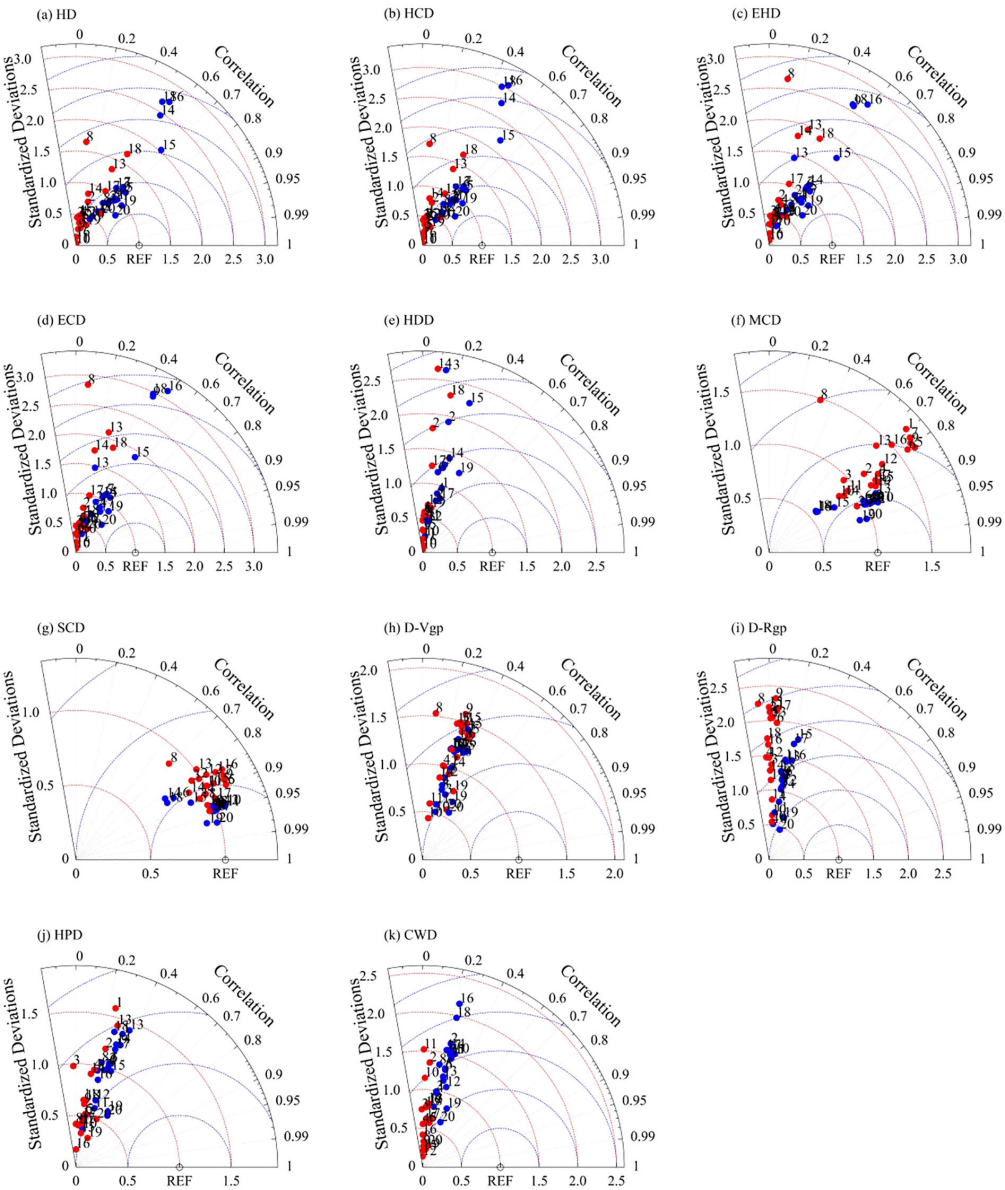
Taylor diagram for 11 extremes indices **(a–k)** of single-rice and early-rice during 1981–2014 for GCM (1-18), AM (19) and IWM (20). (C for observed data, • and • are simulation results without and with deviation correction treatment).

**Figure 5** showed the Taylor diagram which were deviation-corrected of each GCM, AM and IWM for 11 extremes indices during 1981–2014 of single-rice and early-rice. Deviation correction helps increase the correlation coefficient between the simulation results of certain ECIs and the reanalysis data, with the highest correlation coefficient reaching 0.9. **Table 3** showed the root-mean-square error (RMSE) values which were calculated between each GCM, AM, IWM and observations for 11 extreme climate indices during 1981–2014 of single-rice and early-rice. The RMSE of some ECIs also decreased significantly (such as MCD, SCD etc.) (**Table 3**). IWM optimizes the weights by error covariance, which is more flexible than AM, but relies on a complete error covariance estimate. For variables with missing data, such as HDD, AM is more suitable because of its simplicity and stability. In addition, ECIs with low spatial coefficient are still greatly improved after multi-mode ensemble, which shows the effectiveness of the method. Overall, after applying deviation correction to each GCM, the simulation ability of extreme climate events is improved (**Figure 5**; **Supplementary Figure S3**). In terms of RMSE, the simulation ability of each GCM and its multi-model set results for extreme temperature events was also higher than that for extreme precipitation events. In addition, the IWM ensemble results calculated from GCM data with bias correction outperform the AM results in terms of RMSE (**Table 3**; **Supplementary Table S2**). These results suggest that deviation correction can reduce the errors and uncertainties associated with extreme climate events simulated by GCMs to a certain extent, and the IWM ensemble results, obtained using bias corrected GCM data, can better reproduce each extreme climatic event. In cases where the IWM cannot be used of multi-mode collection at certain raster points, the ensemble result from the AM can be used as a suitable alternative.

**Table 3:** Root mean square error between each GCM, the multi-model arithmetic mean, independence weighted mean and observed values for the 11 extreme climate indices during 1981–2014 of single-rice and early-rice, the shaded table is the RMSE of the GCM corrected for deviation, and the non-shaded table is the RMSE of the GCM not corrected for deviation.

GCMs	HD (d)		HCD (d)		EHD (d)		ECD (d)		HDD (°C)		MCD (d)		SCD (d)		D-Vgp (d)		D-Rgp (d)		HPD (d)		CWD (d)	
ACC1	5.0	3.7	4.6	3.6	4.2	3.3	3.7	3.2	4.6	4.7	16.3	5.5	12.2	6.2	12.7	10.8	13.6	9.0	2.8	2.4	2.1	2.3
ACC2	4.9	3.9	4.6	3.8	4.5	4.0	4.0	3.8	8.0	8.0	8.6	5.6	10.2	6.2	12.0	10.3	13.0	7.6	2.3	2.0	2.8	3.0
CAN	5.2	3.8	4.6	3.7	4.5	3.7	3.9	3.5	4.5	4.1	8.4	5.2	10.3	6.1	9.2	8.4	9.0	7.1	2.4	2.0	2.2	2.2
CMC	4.9	3.8	4.5	3.7	4.2	3.6	3.8	3.4	4.3	4.7	9.0	5.4	8.2	6.2	10.0	9.0	9.7	6.5	2.1	2.0	2.0	2.2
ECE1	4.7	4.0	4.3	4.0	4.2	3.7	3.7	3.6	4.5	5.7	14.9	5.2	10.2	6.1	11.1	9.9	12.7	7.5	1.9	2.0	1.9	2.5
ECE2	4.8	4.1	4.4	4.2	4.3	3.9	3.8	3.8	4.6	5.6	13.7	5.6	9.6	6.7	10.8	9.9	12.0	7.6	1.9	2.0	1.9	2.5
ECE3	4.9	4.4	4.4	4.3	4.3	4.0	3.8	3.8	4.5	5.7	15.7	5.2	11.2	6.4	11.2	10.1	12.6	7.5	2.0	2.0	1.9	2.7
FGO	8.6	4.1	8.1	3.9	12.2	3.7	11.3	3.4	22.9	4.4	17.1	5.3	13.8	6.3	14.0	8.7	14.2	7.7	2.1	2.1	1.8	2.7
GFD	4.9	3.8	4.4	3.8	4.3	13.6	3.7	12.9	4.2	28.6	16.6	5.3	11.8	6.1	13.1	10.3	14.3	7.7	1.9	2.3	1.8	2.0
INM1	5.0	4.3	4.4	3.9	4.3	4.0	3.6	3.4	4.1	4.1	7.3	5.3	9.1	6.5	8.8	8.4	6.3	6.0	2.1	2.0	2.6	2.7
INM2	5.0	4.2	4.4	3.8	4.3	3.9	3.7	3.5	4.2	4.3	7.2	5.3	9.2	6.3	9.0	8.5	7.0	6.3	2.0	1.9	3.1	2.7
IPS	4.9	4.0	4.4	3.7	4.3	3.9	3.8	3.5	4.6	4.1	10.3	5.2	10.5	6.5	9.8	9.2	9.6	7.2	2.0	1.8	1.8	2.1
KAC	6.0	4.5	5.8	4.5	8.4	6.0	8.0	5.5	19.9	10.8	11.8	6.4	11.3	6.5	11.9	10.6	13.0	8.8	2.5	2.5	2.1	2.4
MIR	5.3	15.7	4.9	15.2	8.2	4.3	6.9	3.8	11.8	6.1	6.9	7.9	9.7	12.1	9.4	9.4	8.7	7.0	2.1	2.3	2.1	2.9
MPI1	5.1	8.6	4.6	8.7	4.4	6.2	3.8	6.1	4.3	9.0	8.0	6.7	9.2	8.8	12.1	11.5	13.4	10.4	2.0	1.9	1.8	2.8
MPI2	5.0	16.3	4.4	16.6	4.3	12.8	3.6	12.8	4.1	26.7	13.0	8.0	11.3	11.4	9.8	10.3	10.4	8.6	2.1	2.0	1.9	3.8
MRI	4.6	3.7	4.4	3.7	4.7	3.5	4.3	3.4	6.1	4.3	8.0	5.3	7.4	6.0	12.7	10.7	13.5	10.1	2.0	2.2	2.0	2.9
NOR	7.0	16.9	6.7	16.8	7.4	14.4	6.7	13.5	9.8	35.0	7.2	8.1	8.2	11.6	10.6	11.1	11.0	8.2	1.9	2.5	2.1	3.6
AM	4.2	3.8	3.9	3.8	3.7	3.4	3.4	3.3	4.4	5.6	8.1	3.9	7.0	5.2	7.8	7.2	8.5	5.4	1.7	1.5	1.7	1.8
IWM	3.6	2.8	3.5	2.8	3.4	2.7	3.2	2.6	NA	NA	5.3	3.7	6.1	4.5	7.2	6.9	5.9	5.1	1.6	1.4	1.7	1.7

NA signifies the lack of the particular ECI.

### 3.3 Multi model ensemble projections of extreme climate events in the 21st century

**Figure 6:**
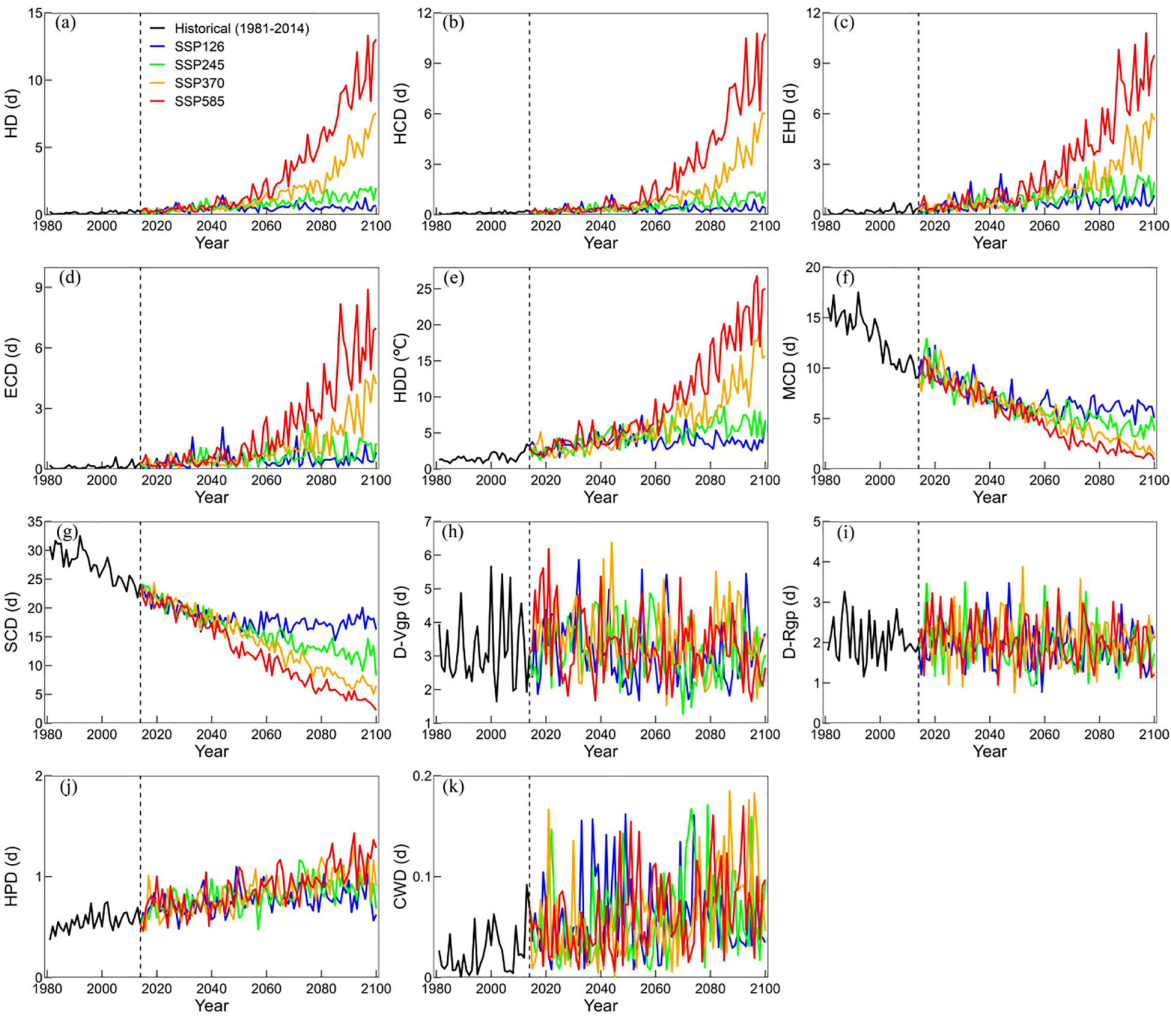
The trends of 11 extreme climate indices (ECIs) **(a–k)** in Zone I during the historical period and 4 future climate scenarios of single-rice and early-rice.

Relative to the historical period, spatial-temporal changes in IWM simulated extreme climate indices under 4 future climate scenarios were shown as **Figure 6**; **Supplementary Figure S4**. Under different climate scenarios for four extreme stress events, the trend change of ECIs was significantly different.

Compare to the historical period, it is obvious that extreme heat stress showed similar change characteristics during the key phenological period of single-rice and early-rice in all zones (**Figures 6a–e**, **Supplementary Figures S5a–e, S6a–e, S7a–e, S8a–e**). Cold stress exhibited a significant decreasing trend across all scenarios in Zone I. This trend is consistent with observed warming patterns and projected climate change impacts, potentially influencing rice cultivation practices and phenological timing in Zone I (**Figures 6f, g**). Drought stress and precipitation stress exhibited slight fluctuations overall, particularly HPD and CWD in Zone I (**Figures 6h–k**). The intensity of extreme heat stress and drought stress in Zone II is stronger than Zone I (**Supplementary Figures S5a–e, S5h, i**) and intensity of cold stress is lower than it, but the precipitation stress showed a more evident intensity of single-rice and early-rice (**Supplementary Figures S5j,k**). In general, the extreme heat stress in Zone III was severe (**Supplementary Figures S6a–e**), the cold stress in Zone IV is the longest (**Supplementary Figures S6f,g**), the drought stress fluctuation is not obvious (**Supplementary Figures S6h, i, S7h, i, S8h, i**), and the precipitation stress displayed an upward trend in Zone III, Zone IV and Zone V (**Supplementary Figures S6j, k, S7j, k, S8j, k**).

Regarding the trends of 11 ECIs in Zone III of late-rice, the variation of HD, HCD appeared to be more pronounced compared to EHD, ECD and HDD of extreme heat stress (**Supplementary Figures S4a–e**). The duration of MCD and SCD varied across regions, with Zone III, Zone IV, and Zone V exhibiting extended periods (**Supplementary Figures S4f, g, S9f, g, S10f, g**). The drought stress of late-rice exhibited slight temporal fluctuations, with distinct stress intensity patterns observed during D-Rgp and D-Vgp (**Supplementary Figures S4h, i, S9h, i, S10h, i**). Precipitation stress all showed a slight upward trend in Zone III, Zone IV and Zone V under 4 different climate change scenarios (**Supplementary Figures S4j, k, S9j, k, S10j, k**).

**Figure 7:**
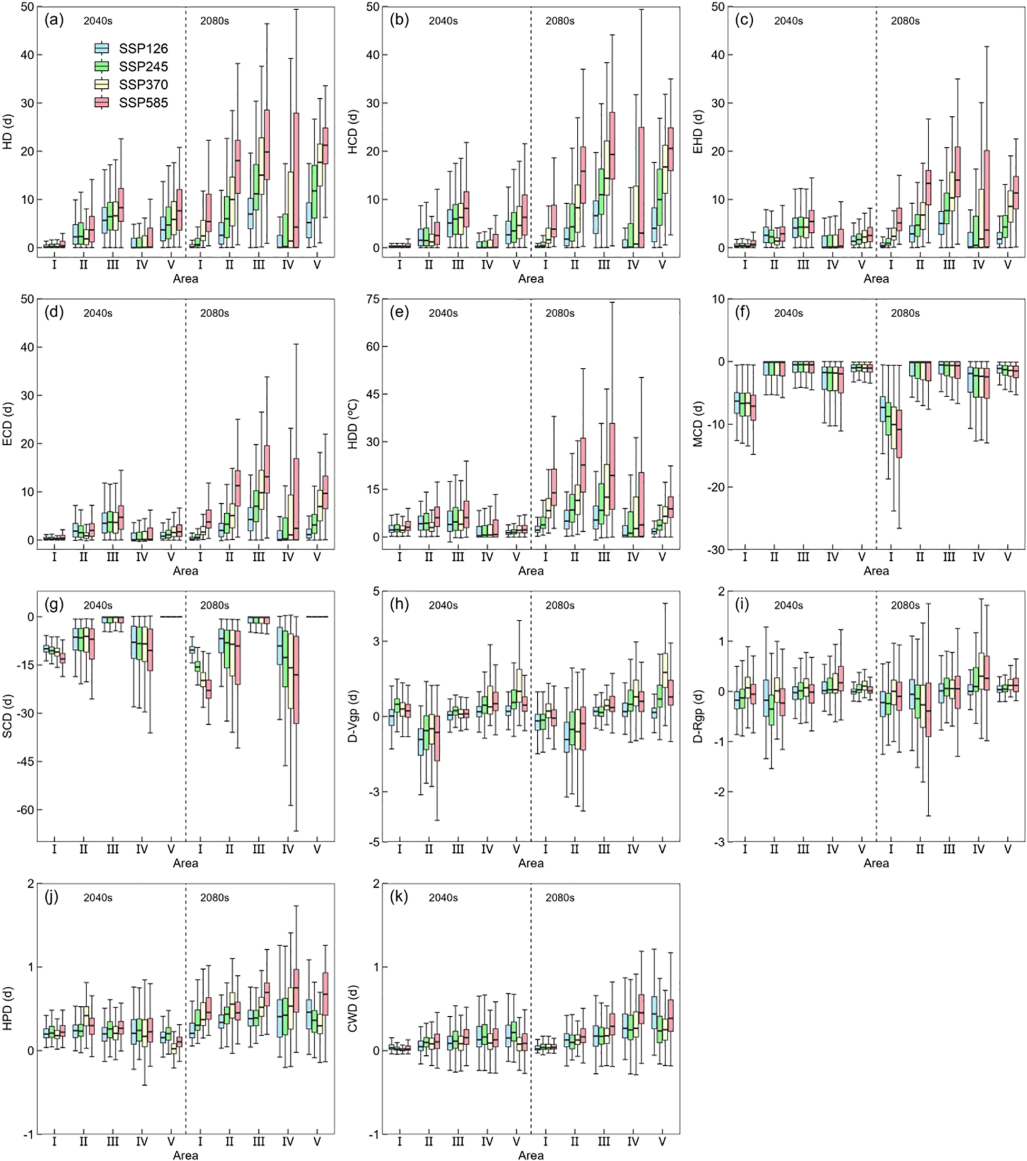
The changes in the 11 extreme climate indices (ECIs) **(a–k)** in 2040s and 2080s under the 4 future climate scenarios compared to the historical periods of single-rice and early-rice.

In the 2080s, extreme heat stress duration for single-rice and early-rice was higher than in the 2040s, especially in Zone III, Zone IV and Zone V (**Figures 7a–e**). Additionally, severe cold stress occurred in Zone I under MCD in the 2040s and in Zone IV under SCD in the 2080s (**Figures 7f, g**). Drought stress has evident impact in Zone II, which may be affected by the climate and location, it is also shows that the impact of less precipitation need to be studied. In general, the change amplitude of the Zone IV is more obvious, the Zone I is more stable (**Figures 7h–k**). The significant changes of extreme heat stress and cold stress index are consistent with the trend of future climate warming, which verifies the rationality of ECIs. Similarly, the high emissions scenario brings more dramatic climate fluctuations. For late-rice, the duration of extreme heat stress in Zone IV is shorter, but the increasing trend of Zone III and Zone V in the 2080s is more obvious than that in the 2040s (**Supplementary Figures S11a–e**). MCD showed a significant descend change under 2080s than 2040s, while SCD showed descend trend in Zone IV (**Supplementary Figures S11f, g**). The performance of drought stress and precipitation stress in Zone V is more obvious. In contrast, Zone III and Zone IV show more modest changes, with Zone IV generally showing the least change (**Supplementary Figures S11h–k**). Overall, the ECIs changes in different zones will be more drastic under the high emission scenario.

**Figure 8:**
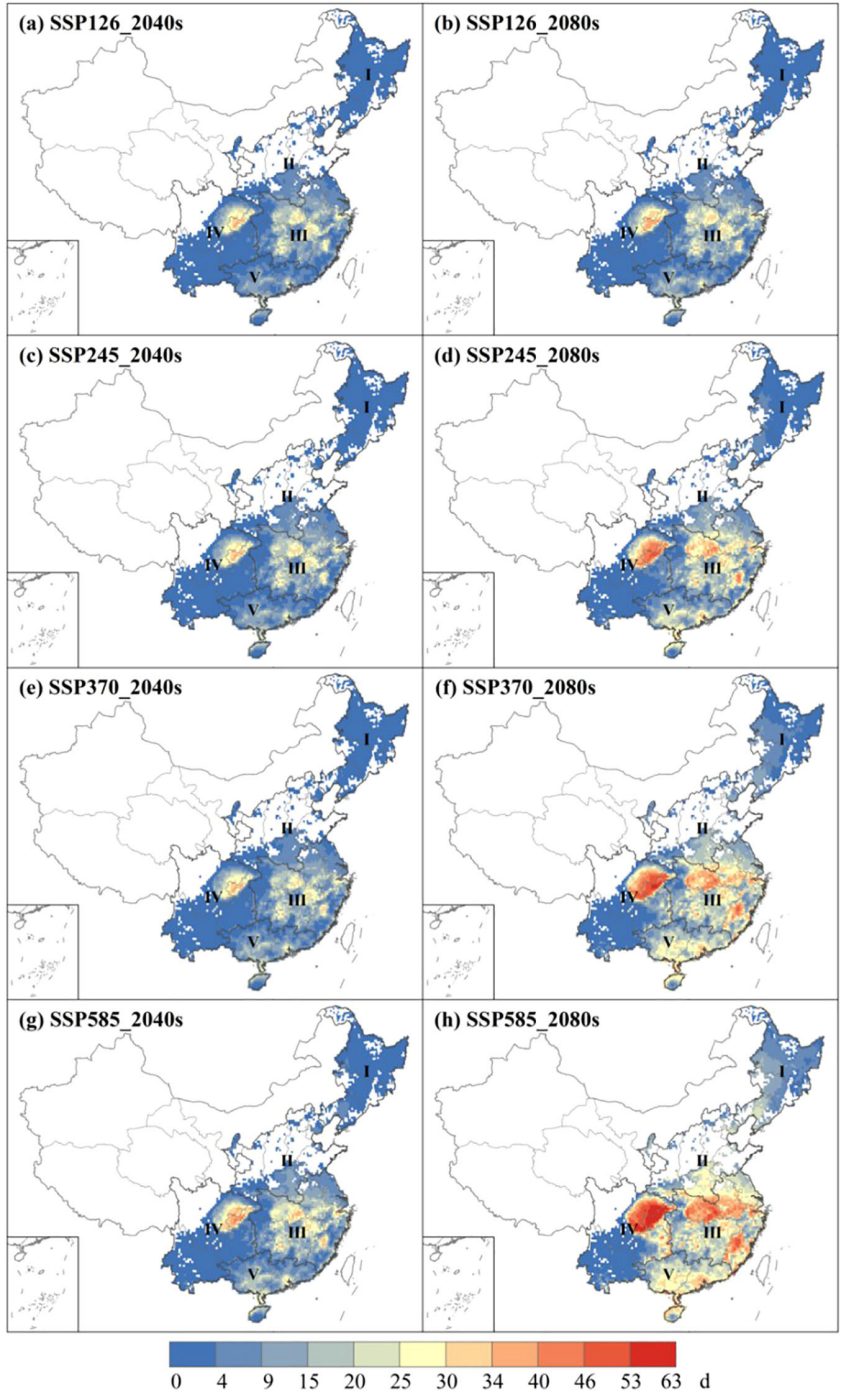
The spatial distribution of HD at 2040s **(a, c, e, g)** and 2080s **(b, d, f, h)** under 4 future climate scenarios of single-rice and early-rice.

Focusing on single-rice and early-rice, it can be observed that under 2040s the main extreme heat stress observed in the central of Zone III and the northeastern of Zone IV (**Figures 8a, c, e, g**). There is a significant increase in the strength of HD (**Figures 8b, d, f, h**), particularly in the southern of Zone V in SSP585 under 2080s (**Figures 8h**), with a linear increase in the intensity of HD as the social emission scenarios. The average number days of HD in four scenarios is 6.1 days (SSP126), 9.6 days (SSP245), 13.3 days (SSP370), 17.8 days (SSP585), respectively, which spatial distribution and duration intensity of HCD, EHD, ECD exhibit similar patterns to HD (**Supplementary Figures S12–S14**). However, the average number days of HDD differs notably under 2080s is 7.4 days (SSP126), 10.5 days (SSP245), 14.3 days (SSP370), 21.4 days (SSP585), respectively (**Supplementary Figures S15b, d, f, h**). Global warming has become the dominant driver of extreme heat stress in rice production, with critical impacts concentrated during flowering and grain-filling stages.

For cold stress of single-rice and early-rice, the variation trend between MCD and SCD showed a sharp decline. The severe regions with severe cold stress were concentrated in western of Zone IV and dispersed in Zone I and eastern of Zone III (**Supplementary Figure S16, S17**). Due to the climatic change, the maximum number of days with MCD under 2040s is projected to be 109.4 days (SSP126). By the 2080s, the minimum number of days with MCD is 72.8 days, which is about two-thirds of the number projected for the 2040swhich is about two-thirds of the number projected for the 2040s (SSP585) (**Supplementary Figures S16a, h**). SCD has a wider spatial extent of cold stress than MCD, mainly affecting Zone IV and Zone I, which also reflects the potential influence regions of future temperature changes.

Regarding the distribution of D-Vgp, drought stress is more severe in the southern region compared to the northern region and in western of Zone II is also affected by drought stress (**Supplementary Figure S18**). Regarding the distribution of D-Rgp, the main affected region is Zone II, northern of Zone III and eastern of Zone IV, while lower drought stress is observed in Zone I and southern of Zone V. The maximum number of days with D-Rgp in these zones is 14.8 days (Zone II), 9.7 days (Zone III) and 9.7 days (Zone IV) under 2040s, which is 14.3 days (Zone II), 10.0 days (Zone III) and 10.3 days (Zone IV) under 2080s (**Supplementary Figure S19**).

Regarding the distribution of HPD, the overall stress intensity is lower than in historical periods, which is consistent with the pattern of global climate change (**Supplementary Figure S20**).Regarding the distribution of CWD, the duration is higher in Zone III, Zone IV and Zone V than the other two zones, the maximum duration is 2.5 days (Zone III), 30.5 days (Zone IV) and 5.3 days (Zone V) under 2040s, which is 2.9 days (Zone III), 33.0 days (Zone IV) and 6.9 days (Zone V) under 2080s (**Supplementary Figure S21**), which indicating the limited effect of extreme precipitation in the future.

**Figure 9:**
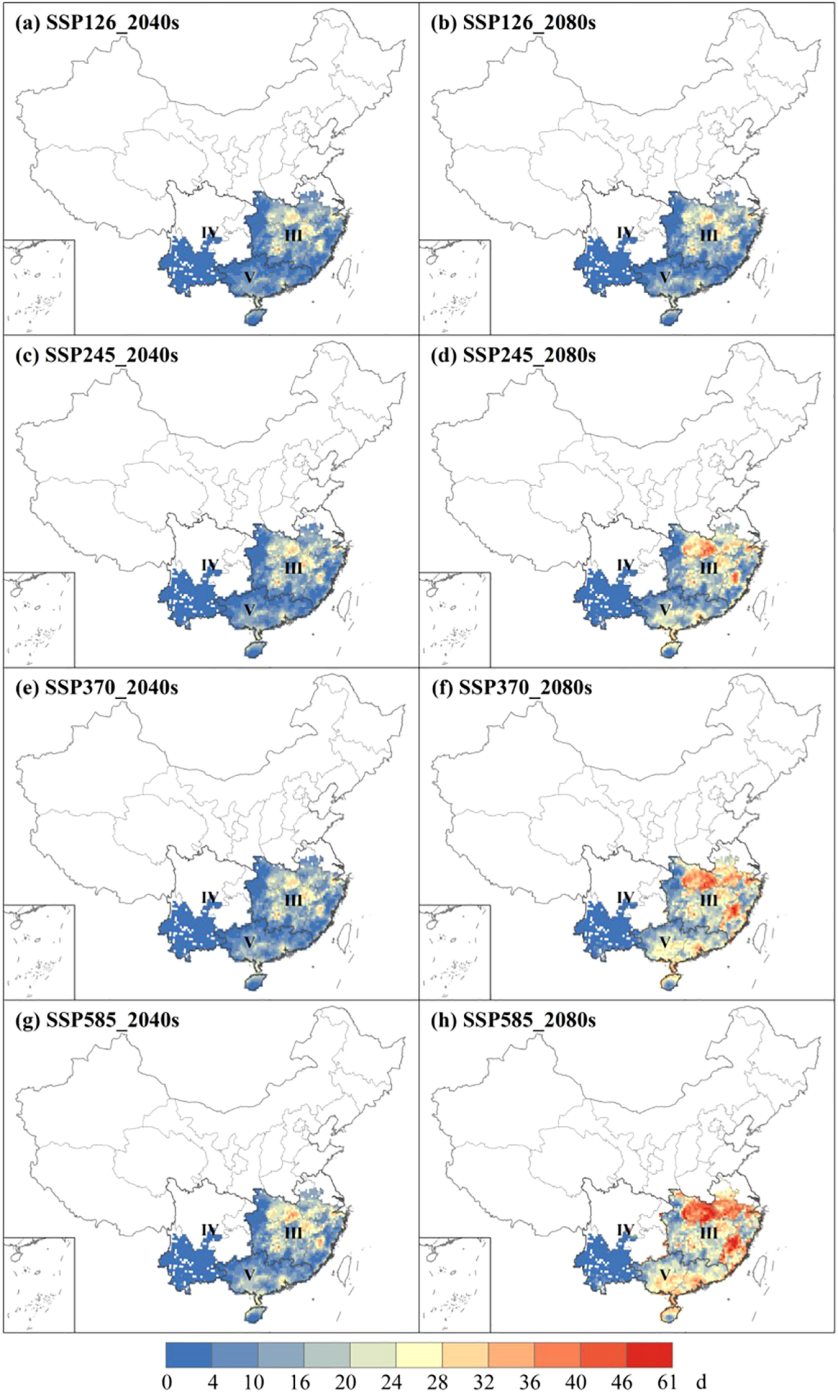
The spatial distribution of HD at 2040s **(a, c, e, g)** and 2080s **(b, d, f, h)** under 4 future climate scenarios of late-rice.

For extreme heat stress of late-rice, there is an evident increasing trend in Zone III and Zone V in two periods. Zone III is the main region affected by HD, with average number of days is 12.4 days (SSP126), 17.5 days (SSP245), 21.8 days (SSP370) and 25.9 days (SSP585) projected for the 2080s (**Figure 9**). The spatial distribution and intensity duration of HCD, EHD and ECD are similar to HD (**Supplementary Figures S22, S23**). Compared to basis line, this represents a rise of 4.9 days (2040s) and 12.4 days (2080s) in SSP585 (**Supplementary Figure S24h** ). As for the intensity of HDD, severe stress is observed in Zone III. Compared to basis line, this represents a rise of 8.0 °C (SSP126), 11.1 °C (SSP245), 14.7 °C (SSP370) and 14.9 °C (SSP585) under 2080s, respectively (**Supplementary Figure S25**). It is evident that rice will be significantly stressed by extreme heat stress in the future.

Compared to baseline, the difference in cold stress between MCD and SCD is evident. The value of MCD is 1.2 days (SSP126), 1.1 days (SSP245), 1.0 days (SSP370), 0.9 days (SSP585) under 2040s, and in the 2080s, the variation is 0.9 days (SSP126), 0.4 days (SSP245), 0.05 days (SSP370), -0.2 days (SSP585) respectively (**Supplementary Figure S26**). The value of SCD is -20.0 days (SSP126), -20.2 days (SSP245), -20.3 days (SSP370), -20.7 days (SSP585) under 2040s and -20.3 days (SSP126), -21.5 days (SSP245), -22.4 days (SSP370), -22.9 days (SSP585), respectively, which indicates a significant difference compared to the baseline (**Supplementary Figure S27**).

Severe drought stress is mainly concentrated in the southern of Zone IV, Zone V in D-Vgp, as well as northern of Zone III and southern of Zone V. These indicate severe stress in all scenarios under the 2040s in D-Vgp, which is consistent with the drought stress projected for the 2080s. And there is an evident change of D-Rgp (**Supplementary Figure S28**). The change of D-Rgp compared to basis line is -10.8 days (SSP126), -10.7 days (SSP245), -10.7 days (SSP370) and -10.7 days (SSP585) under 2040s and -10.8 days (SSP126), -10.7 days (SSP245), -10.6 days (SSP370) and -10.7 days (SSP585) under 2080s (**Supplementary Figure S29**), which indicates a decreasing tend. This indicates the effects of extreme drought events on different phenological stages of late-rice in the future.

Compared to the baseline, there is a difference in precipitation between HPD and CWD. The extreme precipitation stress occurs in Zone IV, with a maximum duration of 9.7 days (SSP126), 10.1 days (SSP245), 9.8 days (SSP370) and 10.1 days (SSP585) under 2040s. In the 2080s, the stress becomes more evident than the 2040s, and the most intense stress is still observed in Zone IV of HPD (**Supplementary Figure S30**). In terms of the spatial distribution of CWD, it is concentrated in the western of Zone IV and the northern of Zone V. The average number days of CWD is 0.8 days (Zone III), 1.9 days (Zone IV), 1.3 days (Zone V), indicating a significant difference among the three zones in SSP585 (**Supplementary Figure S31h**).

## 4 Discussion

### 4.1 Performance of bias correction and multi-model ensemble

Related studies used global and regional climate models to assess the change in extreme climate events and possible impacts as global warming continues (Pan et al., 2022; Shiru et al., 2022; Zamani et al., 2020). In the future, climate change is expected to exacerbate changes in precipitation and temperature, especially the occurrence of extreme climate events, potentially increasing rice’s exposure to climate-related hazards. Generally, extreme climate events greatly impact rice yield and quality, particularly during the sensitive stages of rice development (Li et al., 2015b; Chen et al., 2016). Accurate and quantitative predictions of extreme climate risks can provide information to agricultural producers and policymaker to mitigate the impacts of extreme climates.

In the study of using GCMs, there are large deviations in the simulation of temperature and precipitation, so it is necessary to use DCM to construct future climate scenarios. The DCM is commonly used method for bias correction of climate models, and it performs well in temperature and precipitation noise reduction in this study. The correlation coefficients, which calculated using GCM data after bias correction, and the reanalyzed data are higher than those without bias correction, which can be better in multi-model ensemble. This occurs because bias correction reduces systematic errors in GCM outputs, such as mean biases and variances, aligning them more closely with reanalyzed data. By adjusting these differences, bias correction improves the reliability of the GCM simulations, leading to stronger correlations with reanalyzed data. Moreover, multi-model ensemble effective reduce the uncertainty of single GCM simulation (Bishop and Abramowitz, 2013). Overall, the IWM approach to ensemble ECIs from GCMs outputs of CMIP6 is superior than AM and has a good application effect in this study. Compared with CMIP5 model, CMIP6 model has been proved to be significantly improved, and the simulation of extreme climate events has also been improved (Eyring et al., 2016). This study provides more detailed spatial scenarios of future climate extremes in rice-growing regions of China than previous studies, and uses GCM to participate in multi-mode simulation of four SSPs to reduce the simulation uncertainty caused by the using fewer SSP or GCM (Lee et al., 2021).

### 4.2 Extreme temperature stress affecting rice production

Most of the previous studies focused on a single extreme climate event, or the combination of extreme heat stress and drought stress, extreme heat stress and cold stress, but there were few studies on the evaluation of extreme climate stress at the combined phenological stage in China’s rice growing areas. This study provides a comprehensive assessment of the risk to rice growth based on all stages from transplanting to maturity with global warming. We defined four extreme climate indices, including extreme heat stress, cold stress, drought stress and precipitation stress, through two climate variables, temperature and precipitation (Auffhammer et al., 2012; Schleussner et al., 2018; Tao et al., 2013b; Xiao and Song, 2011).

Extreme temperature stress, encompassing both heat and cold stress events, has shown significant correlations with global warming patterns. Extreme heat stress, characterized by temperatures above a critical threshold, causes irreversible damage to crop growth and development (Wahid et al., 2007). Generally, rice responses differently to high temperatures depending on the stage of their development, and the most affected period usually occurs from heading to maturity. Based on this, we determined more suitable extreme heat stress indices for rice growth, taking into account phenological information and critical crop temperatures thresholds. During critical growth stages of rice such as booting and heading, sustained high temperatures exceeding 35°C can severely impact rice development. Such thermal stress may induce multiple physiological disorders including incomplete panicle differentiation, significant reduction in pollen viability, and fertilization failure that ultimately leads to the formation of empty grains (Huang et al., 2017). The CMIP6 models project a significant increase in the frequency and duration of extreme heat stress in rice-growing areas in China, particularly in Zone III and Zone V (**Supplementary Figures S4, S8**), compared to the baseline. The conclusion is consistent with the enhancement of extreme heat stress for rice caused by global warming observed in previous studies (Ding et al., 2022; Guo et al., 2021; He et al., 2018; Ke and Wen, 2009). This study spanned a long period from 2015 to 2100 and utilized ECIs specifically defined through rice phenological growth stages. Although the time periods we used to define and analyze heat stress differ from previous studies, these results remain scientifically reliable and provide valuable references (Xiong et al., 2016; Chou et al., 2021). In addition, the extreme heat stress of rice growth is still very serious under the background of future climate change. Therefore, the influence of extreme heat stress on rice yield and the improvement of heat resistance of rice varieties should be emphasized in the follow-up research. In regions with adequate resource availability, implementation of supplemental irrigation and optimized fertilizer application should be prioritized to mitigate adverse impacts on rice productivity and yield stability. Cold events, particularly during the reproductive growth period of rice, can lead to incomplete panicle exertion, high spikelet sterility and cause substantial yield loss (Espe et al., 2017; Lesk et al., 2016; Yamamori et al., 2021). Compared to the historical period, the cold stress duration in Zone I, Zone II and Zone IV of single-rice and early-rice, in Zone IV of late-rice is longer in the 21st century (**Figure 6**; **Supplementary Figures S5, S7, S9**). Under various climate scenarios, the trend of cold stress indicates a significant weakening in the northeast and southwest regions. In contrast, other regions either maintain a low level of cold stress or experience its near disappearance, particularly in the southeast of China. This observation is consistent with previous conclusions that cold stress is diminishing at the provincial scale in the southeastern region of China (Wang et al., 2022). The increase of the duration of cold stress in some areas of rice revealed that in the context of global warming, it is still necessary to adjust sowing date or improve cold tolerance to alleviate the damage caused by cold stress (Moura et al., 2017; Olesen et al., 2011).

### 4.3 Extreme precipitation stress affecting rice production

Drought events and heavy precipitation events represent the two primary extreme precipitation stresses influencing rice production. The occurrence of these events during the rice-growing period is determined based on national extreme event standards and is directly related to the intensity and duration of precipitation (Wang et al., 2018; SL424–2008, 2009). Drought is closely related to precipitation, based on which this study defines the drought stress indices. Rice production is highly water dependent and vulnerable to extreme drought events caused by climate change (Piao et al., 2010). The development of rice roots and aerial parts, along with their physiological and biochemical processes, are constrained by drought. Under such conditions, the rice capacity for water uptake and nutrient acquisition is impaired, resulting in growth inhibition that ultimately compromises yield formation (Guo and Zhang, 2024). The results showed that the extreme drought stress increased significantly of single-rice and early-rice in Zone IV and Zone V, and there was also an increase of late-rice in Zone IV but less in Zone III (**Supplementary Figures S6–S8**). The impact of extreme drought on the vegetative stage of rice in the future will be severe, particularly in northern and southwestern China, which is similar to the results of previous study (Guo et al., 2021). This indicates that there is evident agricultural water pressure in these areas, which will also affect local agricultural development. Therefore, it is possible to mitigate the impact of drought stress by cultivating and planting more drought-resistant rice varieties or by improving the management of farmland water usage, thus alleviating agricultural water pressure in severely drought-affected regions and better adapting to future climate change.

Conversely, excessive precipitation intensity will cause physical damage to rice accompanied by low temperature and weak light, and will also increase the risk of diseases and pests, posing a threat to rice production (Choi et al., 2013). Under different situations, the frequency of extreme precipitation has increased, especially in the southern regions in China. Although rice plants require large amount of water during growth, too much precipitation can lead to a serious loss of rice yield (Panda and Barik, 2021). The results of this study are consistent with previous studies, and there is a strong correlation between increased extreme heat stress and extreme precipitation stress (Myhre et al., 2019). In conclusion, the effects of extreme climate stress can be mitigated by adjusting sowing dates during key growth periods, and by improving rice genes and breeding rice varieties with strong stress resistance. Additionally, adaptive irrigation, fertilization, and other management practices should be implemented in accordance with regional environmental and climatic variations.

### 4.4 Limitations and uncertainties

There were some limitations in this study. Firstly, future interannual changes in climate and occasional extreme events may lead to changes in rice phenology. We defined the extreme climate stress during rice growth period based on historical average phenological data and predicted the extreme climate during future rice-growth periods, but this study did not account for that the potential changes in rice phenology due to climate change, which could lead to some deviation in the prediction results. This study assumes that the future phenological stage is consistent with the historical period, which is still valuable for simulating extreme climate events faced by rice. In general, the result can reasonably reflect the extreme climate events that may arise during future rice-growth. Besides that, there are also uncertainties in the assessment of changes in the extreme climate indices in this study, including the deviation correction methods for the GCM. Therefore, additional comparative evaluations of future climate extremes in rice growing regions in China through more GCMs, different multi-model ensemble methods, and improved bias correction techniques are warranted. In addition, the future calculation of extreme climate stress can pay more attention to the change of rice yield, and further refine the impact of extreme climate stress on rice yield under different future scenarios. Future studies of rice resistance genes are likely to reduce rice vulnerability to extreme climate, thus lowering the risk compared to current estimates. And the combination of multiple factors (e.g. optimization of deviation method, adjustment of sowing date, changes in phenology, rice yield) will be able to better accurately assess the extreme climate stress faced by rice in later study.

## 5 Conclusion

This study conducted a comprehensive assessment of projected extreme climate characteristics and trends under four future scenarios using 18 selected Global Climate Models (GCMs) from CMIP6. By defining 11 Extreme Climate Indices (ECIs), we systematically evaluated potential climate risks in China’s rice-growing regions during the 21st century. Through analyzing differential impacts of temperature and precipitation, distinct heterogeneity patterns of future extreme climate changes were identified across zones. The main findings are as follows:

1. In the 21st century, extreme heat stress intensity is projected to significantly increase under different scenarios, especially in Zone III, Zone IV and Zone V for single-rice and early-rice and Zone III, Zone V for late-rice. Global warming poses a substantial threat to rice production in China, with varying levels of intensity across the entire region. Conversely, cold stress risks for single-rice and early-rice show declining trends due to warming, with late-rice in southern China expected to be rarely face severe cold stress by the late 21st century.
2. Global warming is causing changes in temperature and precipitation patterns, which in turn have an impact on crop productivity. The projected increases in frequency and intensity of extreme precipitation stress are markedly less pronounced compared to extreme temperature stress. In the border region between Zone II and Zone III, which is the North Plain area of China, rice planting is most vulnerable to be affected by drought stress. Regarding extreme precipitation related stresses, southern China consistently demonstrates higher vulnerability than northern regions across all rice systems.

## Data Availability

The source code of Global Climate Models is provided by the Coupled Model Intercomparison Project Phase 6 (CMIP6) (https://aims2.llnl.gov/search/cmip6). The source code of ECMWF Reanalysis v5 (ERA5) is provided by the European Centre for Medium-Range Weather Forecasts (ECMWF) (https://cds.climate.copernicus.eu/). The source code of Rice Phenology Data is provided by the ChinaCropPhen1km dataset (https://figshare.com/) (Luo et al., 2020). The datasets generated and analyzed during the current study are available from the corresponding author on reasonable request.
