# Supplementary material for: Projected future changes in extreme climate indices affecting rice production in China using a multi-model ensemble of CMIP6 projections
Source: Front Plant Sci. 2025 Jul 17;16:1595367. doi: 10.3389/fpls.2025.1595367 (PMC12310666; doi:10.3389/fpls.2025.1595367)
Supplement: Supplementary file 1 [file DataSheet1.docx]

Supplementary Materials

**Projected future changes in extreme climate indices affecting rice production in China using a Multi-model ensemble of CMIP6 projections**

Xinmin Chen^1^, Dengpan Xiao^1,2*^, Yongqing Qi^3^, Zexu Shi^1^, Huizi Bai^4^, Yang Lu^1,2^, Man Zhang^1,2^, Peipei Pan^1,2^, Dandan Ren^5^, Xiaomeng Yin^1,2^, Renjie Li^1,2*^

^1^College of Geography Science, Hebei Normal University, Shijiazhuang 050024, China

^2^Hebei Laboratory of Environmental Evolution and Ecological Construction, Shijiazhuang 050024, China

^3^Key Laboratory for Agricultural Water Resources, Hebei Key Laboratory for Agricultural Water Saving, Center for Agricultural Resources Research, Institute of Genetics and Developmental Biology, Chinese Academy of Sciences, Shijiazhuang 050021, China

^4^Engineering Technology Research Center, Geographic Information Development and Application of Hebei, Institute of Geographical Sciences, Hebei Academy of Sciences, Shijiazhuang 050011, China

^5^School of Resources and Environment, College of Carbon Neutrality, Linyi University, Linyi, China

* Correspondence: Dengpan Xiao, [xiaodp@sjziam.ac.cn;](mailto:xiaodp@sjziam.ac.cn;) Renjie Li, lrjgis@hebtu.edu.cn

**Table S1.** The detail information about location, rice system, climate condition in five rice cultivation zones.

| ​Item | Zone I | ​Zone II | ​Zone III | ​Zone IV | ​Zone V |
| --- | --- | --- | --- | --- | --- |
| ​Location | Northeast China | North China Plain | Middle-Lower Yangtze River Basin | Southwest China | Southern China |
| ​Dominant Crops | Single-rice | Single-rice | Single-rice, Early-rice, Late-rice | Single-rice, Late-rice | Early-rice, Late-rice |
| ​Cropping System | Single cropping | Single cropping | Double or Triple cropping | Double or Triple cropping | Double or Triple cropping |
| ​Climate Type | Temperate | Subtropical | Subtropical | Subtropical | Subtropical |
| ​Annual Mean Temperature (°C) | 1–7 | 9–15 | 15–18 | 16–24 | 17–24 |
| ​Annual Precipitation (mm) | 400–1000 | 580–1000 | 700–1300 | 800–1600 | 1000–2000 |
| ​Annual Accumulated Temperature (>10°C) | 2000–3100 | 2750–4900 | 4500–5500 | 3500–6000 | 6500–8000 |
| ​Proportion of Planting Area (%) | 14.7 | 3.2 | 51.2 | 14.8 | 16.1 |

**Table S2.** Root mean square error between each GCM, the multi-model arithmetic mean, independence weighted mean and observed values for the 11 extreme climate indices during 1981–2014 of late-rice, the shaded table is the root mean square error of the GCM corrected for deviation, and the non-shaded table is the root mean square error of the GCM not corrected for deviation.

| GCMs | HD (d) | | HCD (d) | | EHD (d) | | ECD (d) | | HDD (℃) | | MCD (d) | | SCD (d) | | D-Vgp (d) | | D-Rgp (d) | | HPD (d) | | CWD (d) | |
| --- | --- | --- | --- | --- | --- | --- | --- | --- | --- | --- | --- | --- | --- | --- | --- | --- | --- | --- | --- | --- | --- | --- |
| ACC1 | 1.1 | 1.1 | 0.9 | 1.0 | 1.3 | 1.3 | 1.0 | 1.1 | 1.6 | 1.7 | 5.1 | 3.8 | 15.4 | 12.4 | 8.8 | 8.9 | 15.0 | 14.2 | 1.9 | 1.6 | 1.9 | 1.7 |
| ACC2 | 1.1 | 1.2 | 0.9 | 1.0 | 1.3 | 1.5 | 1.1 | 1.2 | 1.8 | 2.0 | 3.5 | 3.9 | 13.0 | 11.1 | 7.1 | 7.3 | 15.3 | 14.6 | 1.7 | 1.4 | 3.0 | 2.2 |
| CAN | 1.2 | 1.6 | 1.0 | 1.4 | 1.4 | 1.5 | 1.1 | 1.3 | 1.7 | 2.1 | 3.6 | 3.9 | 10.6 | 10.2 | 7.6 | 7.1 | 14.9 | 14.2 | 1.7 | 1.6 | 1.9 | 1.7 |
| CMC | 1.1 | 1.3 | 0.9 | 1.1 | 1.5 | 2.0 | 1.3 | 1.7 | 1.7 | 2.2 | 3.0 | 3.1 | 10.6 | 10.0 | 5.5 | 5.5 | 15.8 | 14.2 | 1.5 | 1.6 | 1.7 | 1.8 |
| ECE1 | 1.1 | 1.2 | 1.0 | 1.1 | 1.4 | 1.6 | 1.1 | 1.3 | 1.7 | 2.0 | 5.4 | 3.1 | 9.5 | 8.6 | 7.2 | 6.5 | 15.5 | 14.6 | 1.2 | 1.3 | 1.2 | 1.6 |
| ECE2 | 1.3 | 1.7 | 1.2 | 1.5 | 1.7 | 2.4 | 1.4 | 2.0 | 2.2 | 3.0 | 4.8 | 2.8 | 10.1 | 10.2 | 7.0 | 6.4 | 15.1 | 14.8 | 1.3 | 1.3 | 1.3 | 1.7 |
| ECE3 | 1.1 | 1.3 | 1.0 | 1.1 | 1.4 | 1.7 | 1.1 | 1.4 | 1.7 | 2.1 | 7.3 | 4.0 | 10.2 | 9.4 | 7.0 | 6.6 | 15.0 | 16.6 | 1.3 | 1.3 | 1.2 | 1.8 |
| FGO | 1.7 | 1.1 | 1.5 | 0.9 | 4.6 | 1.3 | 3.8 | 1.0 | 6.3 | 1.5 | 5.2 | 2.0 | 14.9 | 8.1 | 9.0 | 6.3 | 15.2 | 15.2 | 1.3 | 1.6 | 1.2 | 2.1 |
| GFD | 1.1 | 1.1 | 0.9 | 0.9 | 1.2 | 1.2 | 1.0 | 1.0 | 1.5 | 1.5 | 5.5 | 3.5 | 17.6 | 13.9 | 7.3 | 6.7 | 15.2 | 14.4 | 1.4 | 1.7 | 1.1 | 1.3 |
| INM1 | 1.1 | 1.1 | 0.9 | 0.9 | 1.2 | 1.2 | 1.0 | 1.0 | 1.5 | 1.5 | 3.7 | 4.7 | 11.6 | 10.3 | 5.0 | 5.0 | 16.2 | 14.6 | 1.3 | 1.3 | 3.2 | 1.8 |
| INM2 | 1.1 | 1.1 | 0.9 | 0.9 | 1.2 | 1.2 | 1.0 | 1.0 | 1.5 | 1.5 | 3.5 | 4.4 | 13.5 | 11.3 | 5.1 | 5.1 | 16.1 | 15.2 | 1.5 | 1.2 | 3.9 | 2.0 |
| IPS | 1.1 | 1.2 | 0.9 | 1.0 | 1.2 | 1.2 | 1.0 | 1.0 | 1.5 | 1.5 | 5.0 | 4.0 | 10.0 | 10.6 | 5.3 | 5.3 | 16.0 | 15.6 | 1.3 | 1.3 | 1.0 | 1.4 |
| KAC | 1.4 | 2.3 | 1.2 | 2.0 | 3.9 | 2.1 | 3.4 | 1.9 | 6.9 | 4.4 | 4.3 | 4.1 | 10.5 | 11.6 | 9.1 | 9.0 | 14.6 | 14.4 | 1.7 | 1.7 | 1.8 | 1.9 |
| MIR | 1.6 | 2.4 | 1.4 | 2.0 | 3.2 | 4.4 | 2.5 | 3.5 | 4.4 | 6.3 | 2.9 | 3.6 | 9.8 | 11.1 | 6.4 | 7.1 | 15.4 | 15.0 | 1.5 | 1.4 | 2.2 | 2.1 |
| MPI1 | 1.1 | 1.2 | 0.9 | 1.0 | 1.2 | 1.3 | 1.0 | 1.0 | 1.5 | 1.5 | 3.9 | 2.6 | 12.8 | 10.5 | 8.3 | 8.0 | 14.7 | 24.4 | 1.3 | 1.3 | 1.1 | 1.7 |
| MPI2 | 1.1 | 1.1 | 0.9 | 0.9 | 1.2 | 1.2 | 1.0 | 1.0 | 1.5 | 1.5 | 6.8 | 4.5 | 14.7 | 11.9 | 7.2 | 7.3 | 15.6 | 22.7 | 1.3 | 1.3 | 1.5 | 2.3 |
| MRI | 1.2 | 1.5 | 1.0 | 1.4 | 1.3 | 1.4 | 1.0 | 1.1 | 1.5 | 1.6 | 3.3 | 2.9 | 9.9 | 9.0 | 11.7 | 10.0 | 14.0 | 18.0 | 1.3 | 1.8 | 1.3 | 2.1 |
| NOR | 2.9 | 3.9 | 2.5 | 3.5 | 3.1 | 4.2 | 2.6 | 3.7 | 3.8 | 5.9 | 2.6 | 3.2 | 9.7 | 10.7 | 8.1 | 8.2 | 15.1 | 16.3 | 1.4 | 1.6 | 1.7 | 2.4 |
| AM | 1.0 | 1.0 | 0.9 | 0.9 | 1.2 | 1.2 | 1.0 | 1.0 | 1.6 | 1.6 | 2.9 | 2.4 | 8.2 | 7.0 | 4.9 | 4.8 | 14.2 | 10.9 | 1.1 | 1.0 | 1.0 | 1.1 |
| IWM | 1.0 | 0.9 | 0.9 | 0.9 | 1.1 | 1.1 | 1.0 | 1.0 | NA | NA | 2.1 | 1.6 | 6.9 | 6.3 | 4.6 | 4.6 | 11.9 | 9.8 | 1.0 | 1.0 | 0.9 | 1.0 |


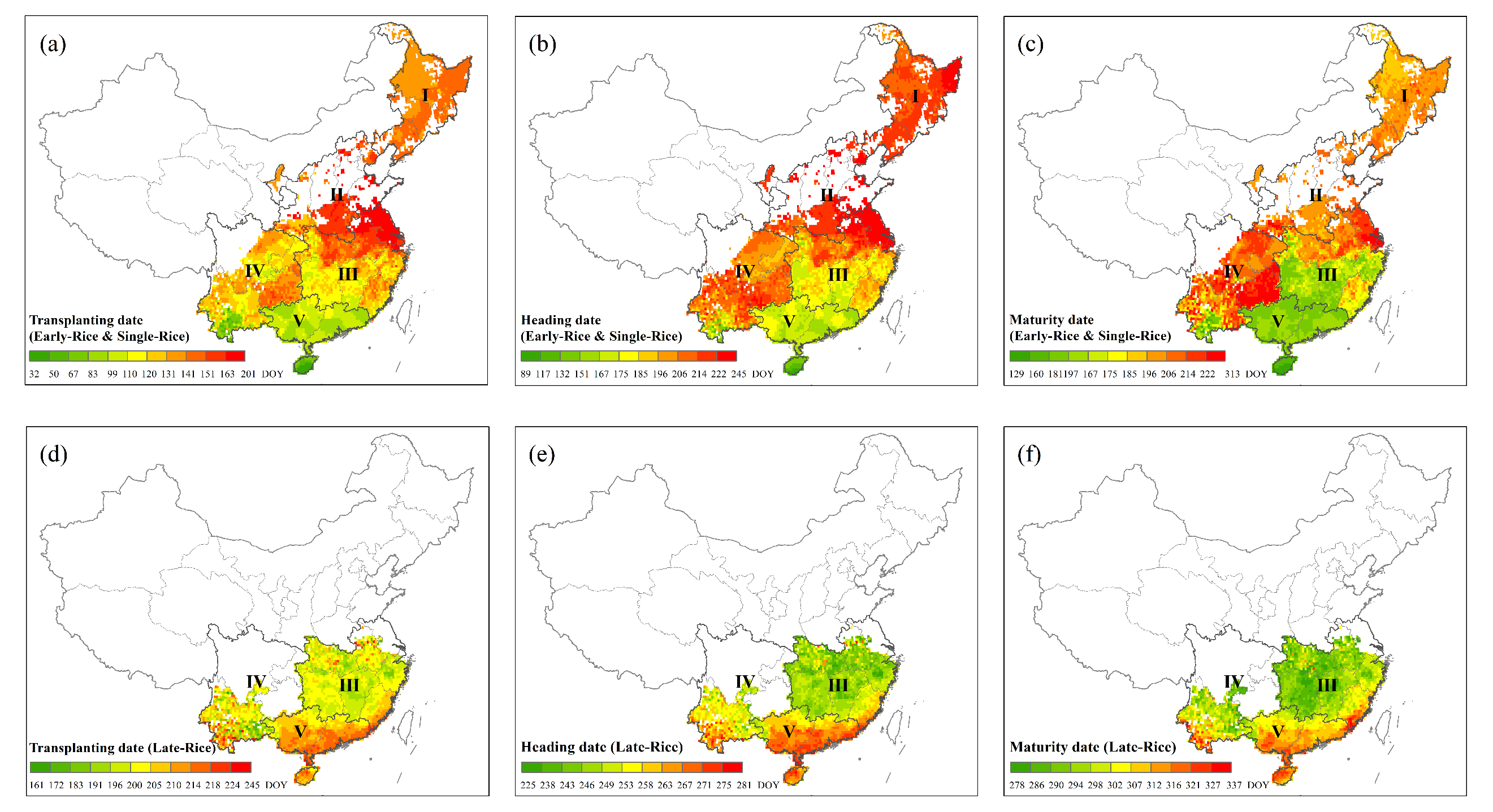


**Figure S1.** Day of year about key phenological period of spatial in rice-growing area is depicted. Three period for single-rice and early-rice is transplanting date (a), heading date (b) and maturity date (c) and for late-rice is transplanting date (d), heading date (e) and maturity date (f).


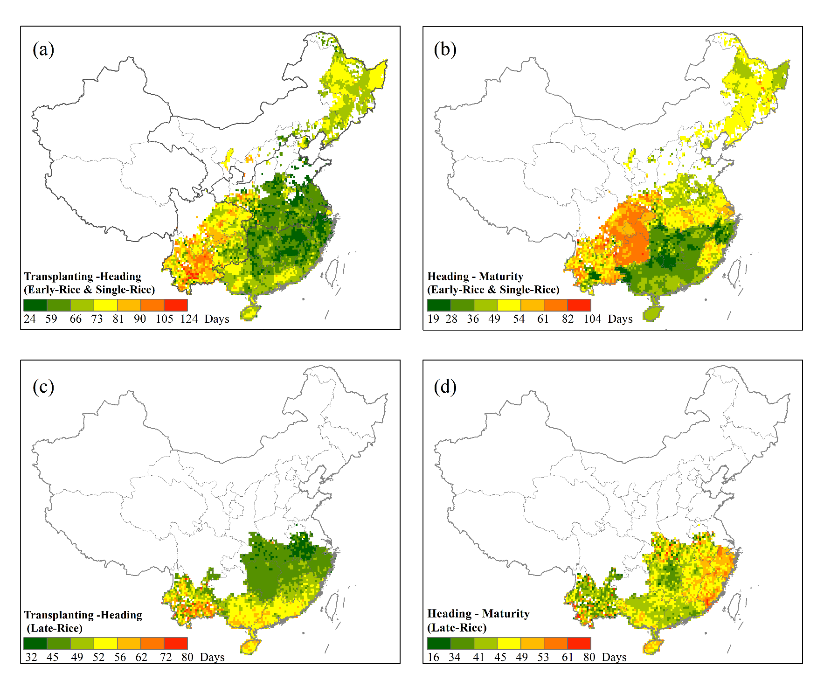


**Figure S2.** Days of phenological period of spatial in rice-growing area is depicted. The period for single-rice and early-rice is transplanting-heading (a), heading-maturity (b) and for late-rice is transplanting- heading (c), heading-maturity (d).


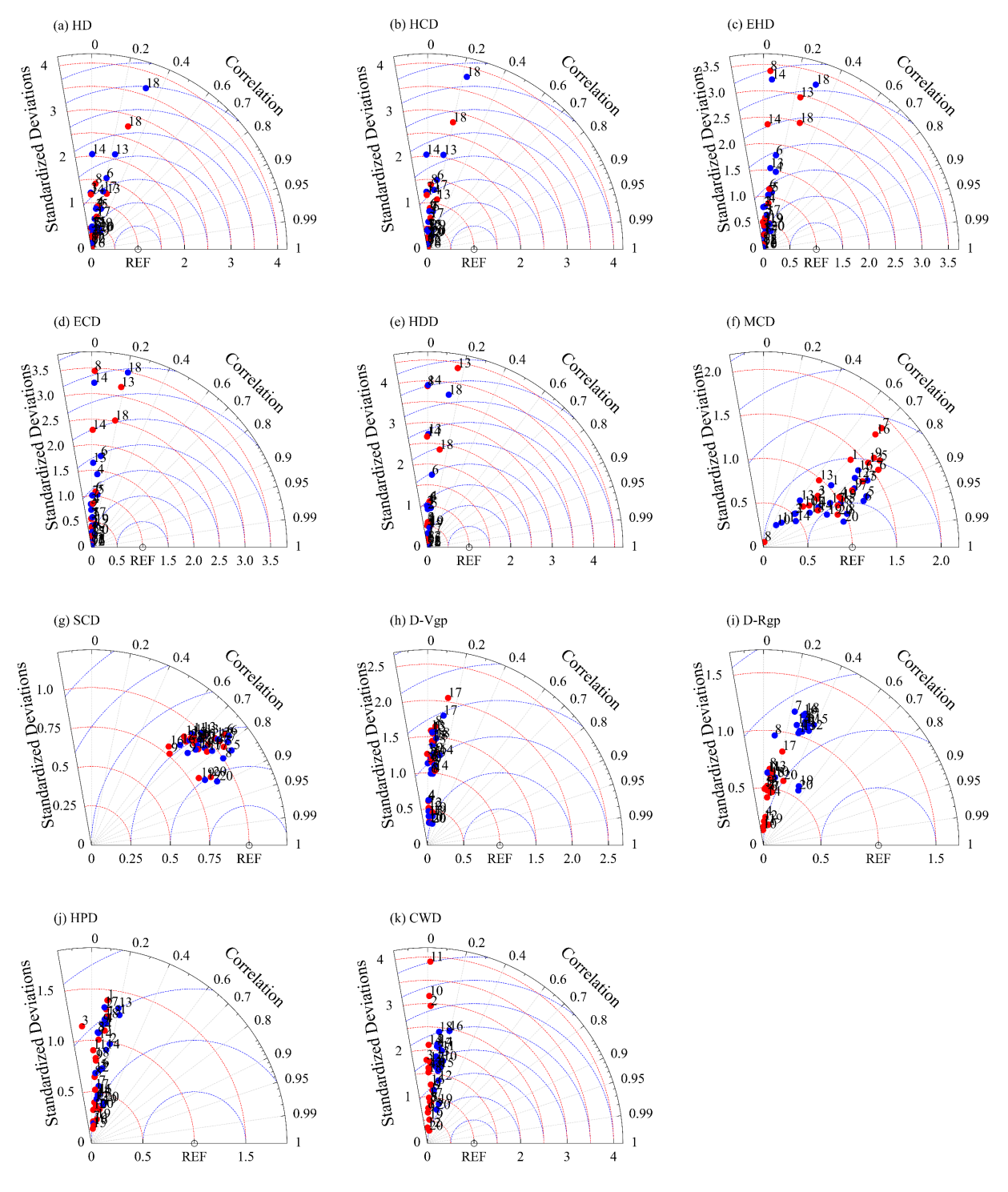


**Figure S3.** Taylor diagram for 11 extremes indices 1981–2014 for GCM (1-18), AM (19) and IWM (20) of late-rice. (○ for observed data, • for simulation results without deviation correction treatment, and • for simulation results with deviation correction treatment).


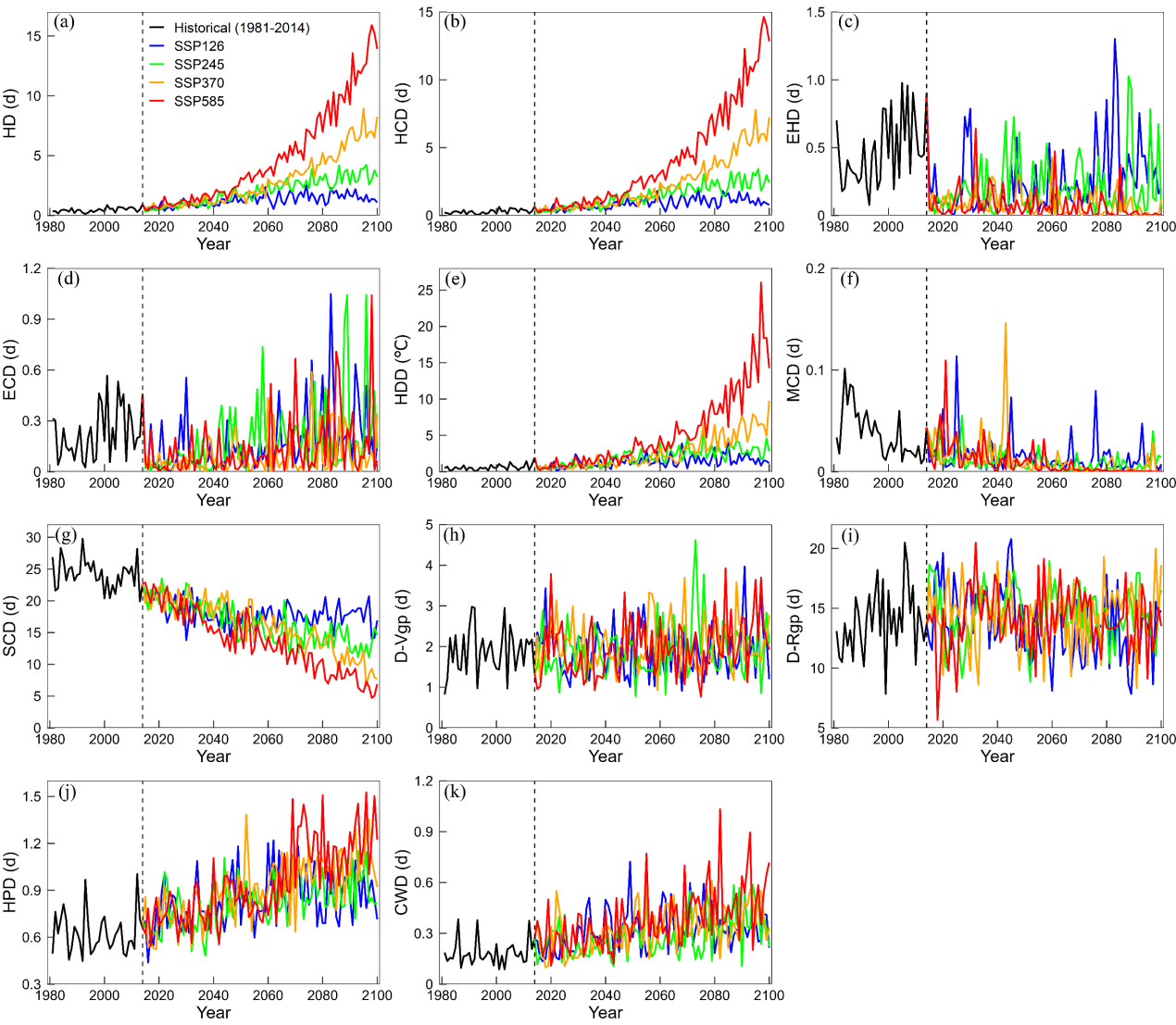


**Figure S4.** The trends of 11 extreme climate indices (ECIs) in Zone Ⅲ during the historical period and 4 future climate scenarios of late-rice.


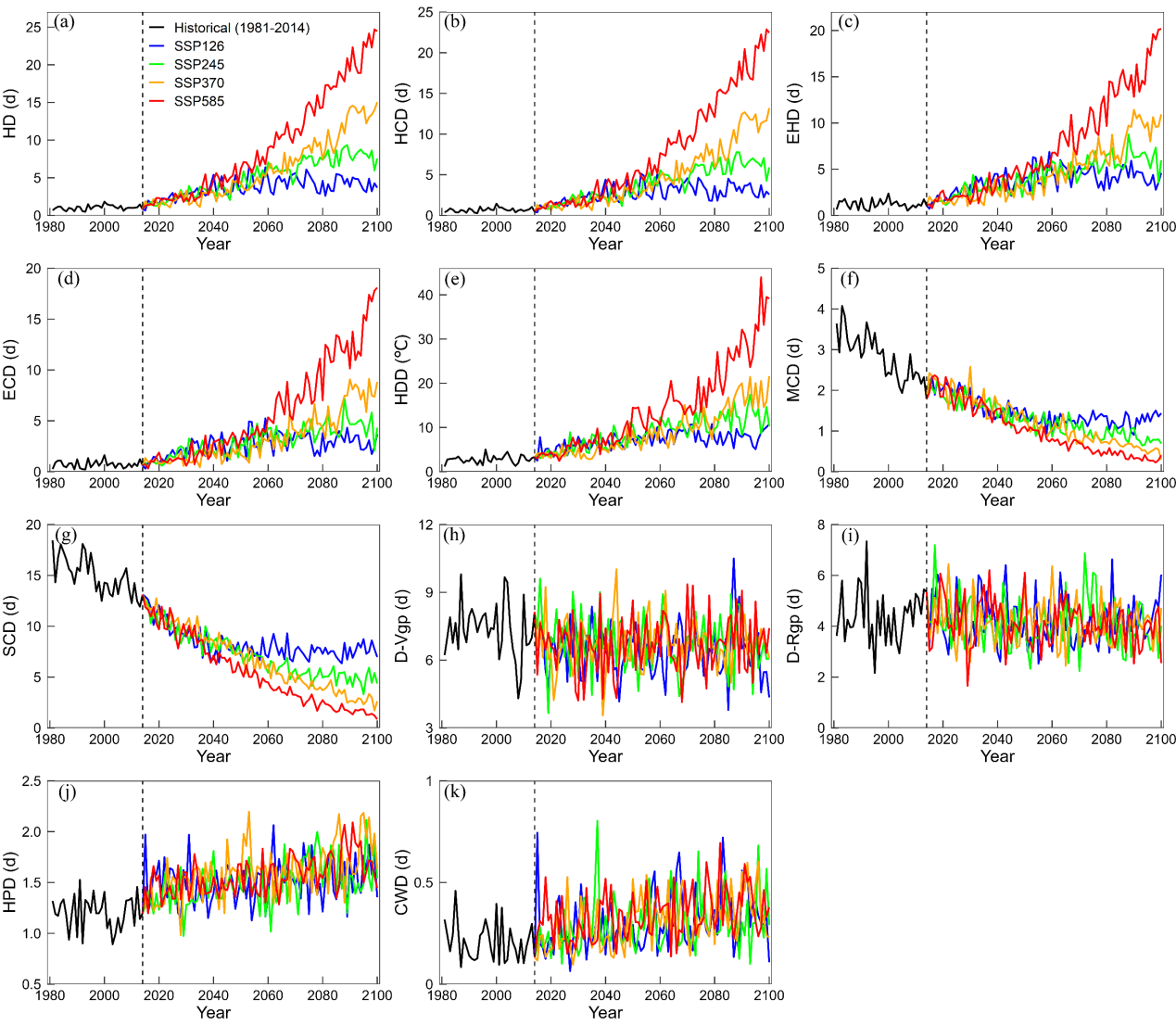


**Figure S5.** The trends of 11 extreme climate indices (ECIs) in Zone Ⅱ during the historical period and 4 future climate scenarios of single-rice and early-rice.


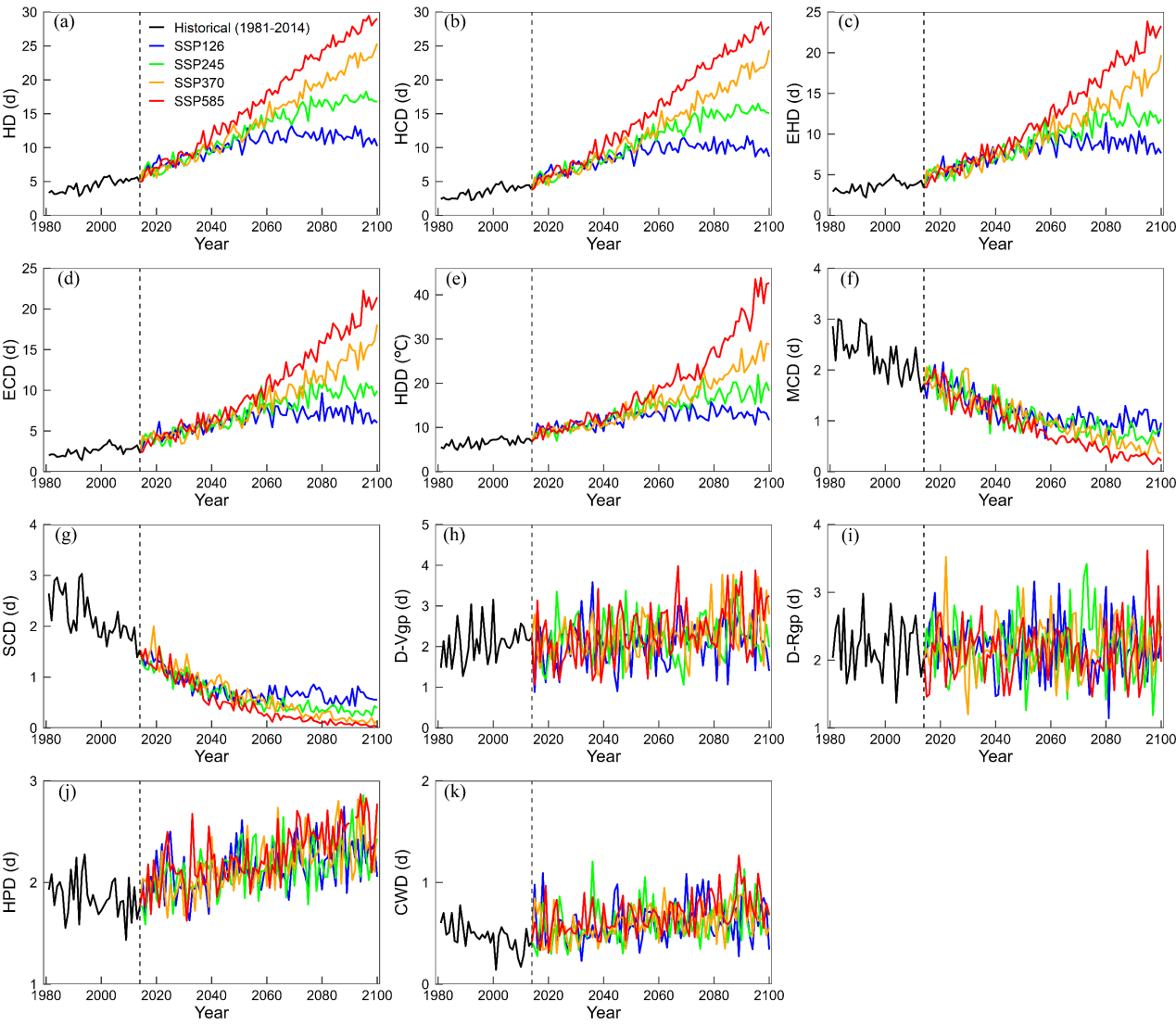


**Figure S6.** The trends of 11 extreme climate indices (ECIs) in Zone Ⅲ during the historical period and 4 future climate scenarios of single-rice and early-rice.


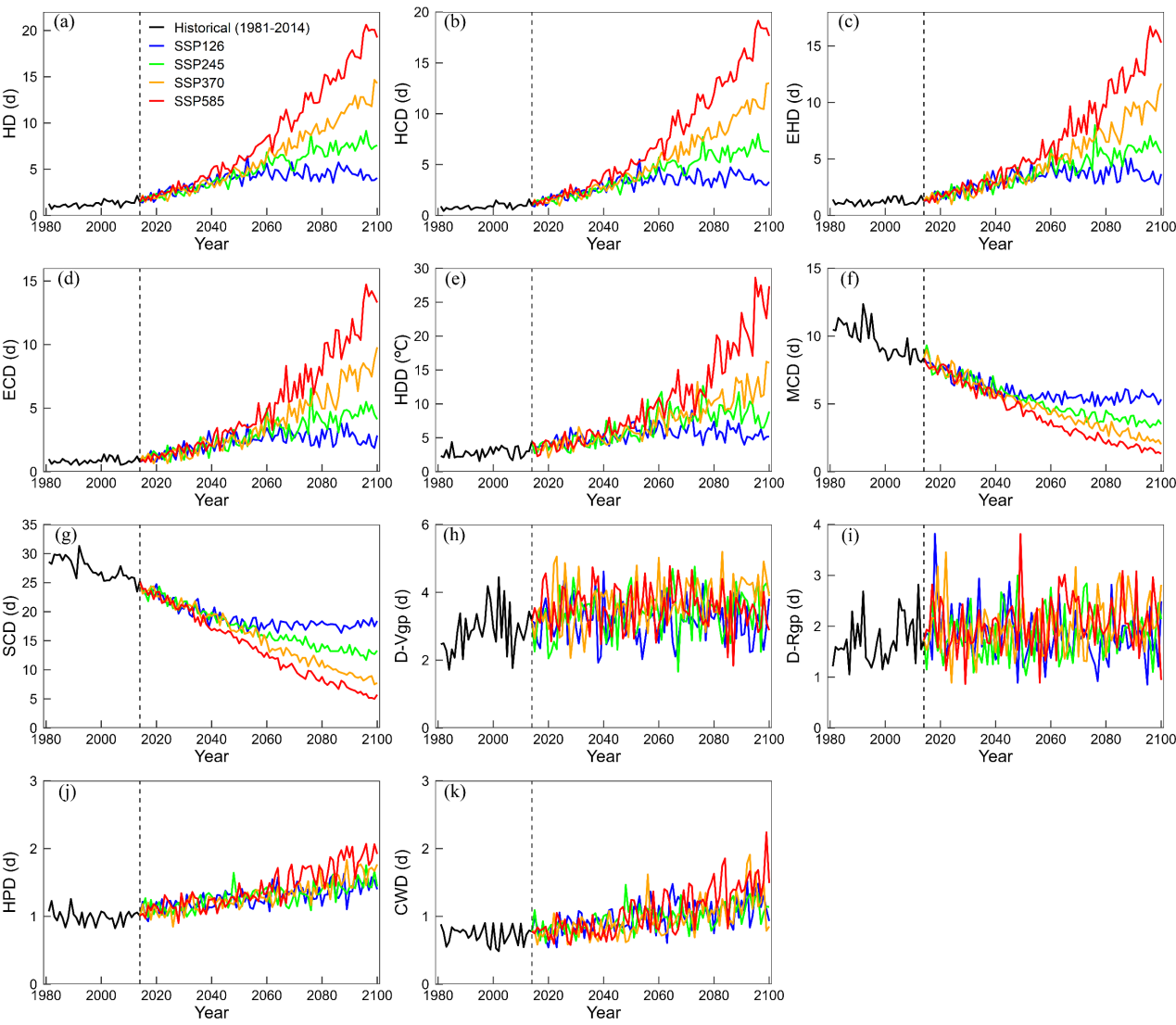


**Figure S7.** The trends of 11 extreme climate indices (ECIs) in Zone Ⅳ during the historical period and 4 future climate scenarios of single-rice and early-rice.


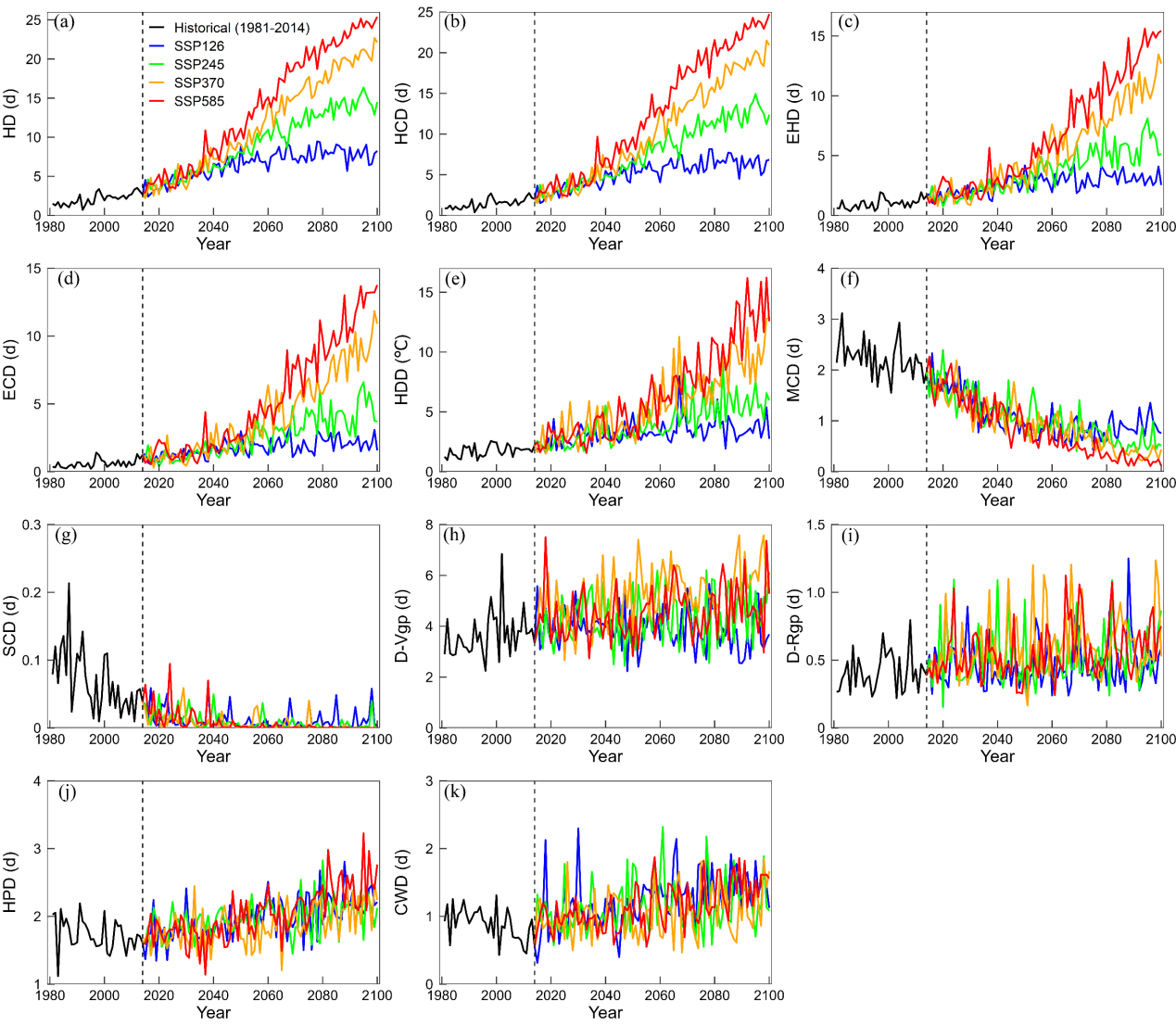


**Figure S8.** The trends of 11 extreme climate indices (ECIs) in Zone Ⅴ during the historical period and 4 future climate scenarios of single-rice and early-rice.


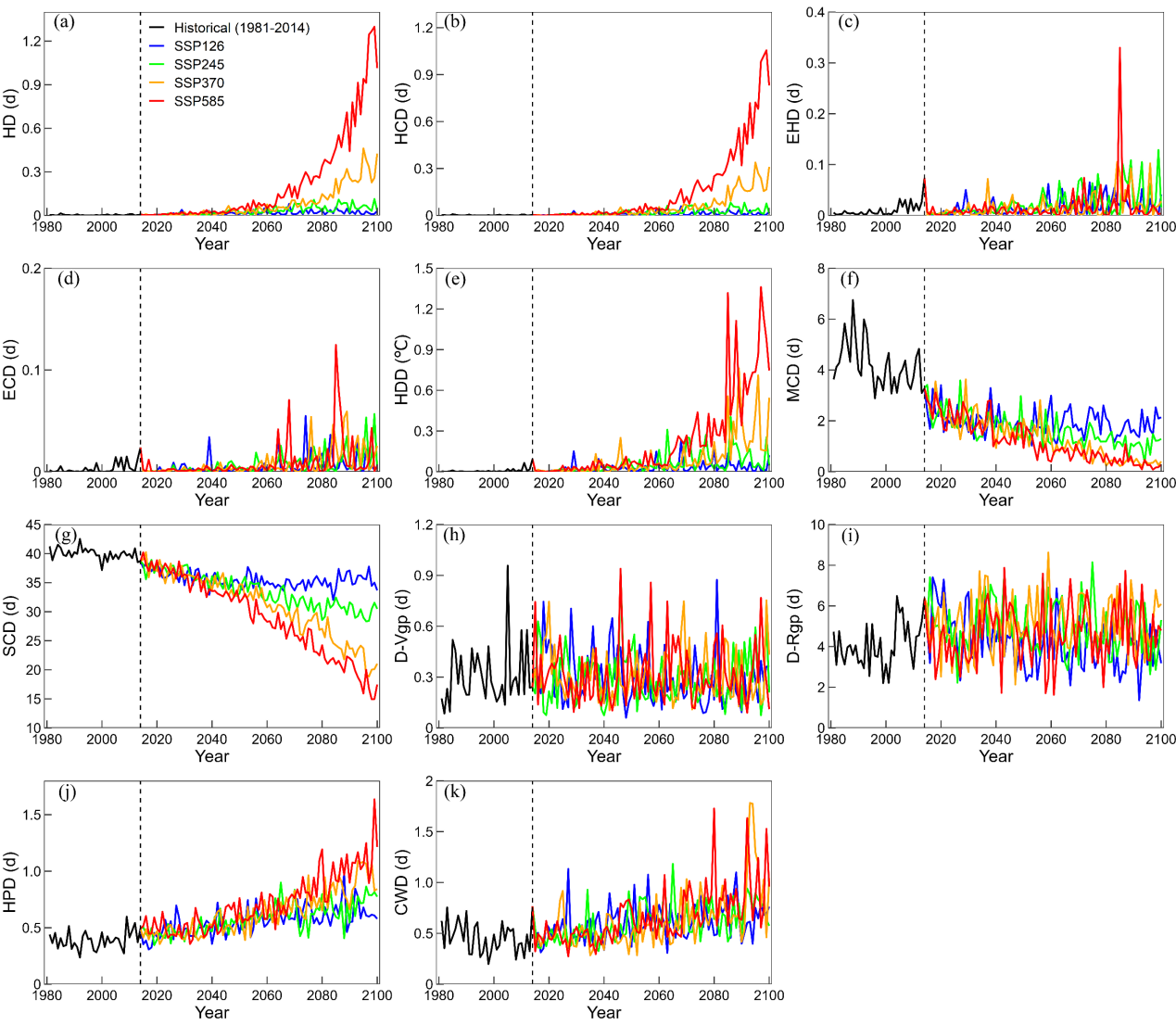


**Figure S9.** The trends of 11 extreme climate indices (ECIs) in Zone Ⅳ during the historical period and 4 future climate scenarios of late-rice.


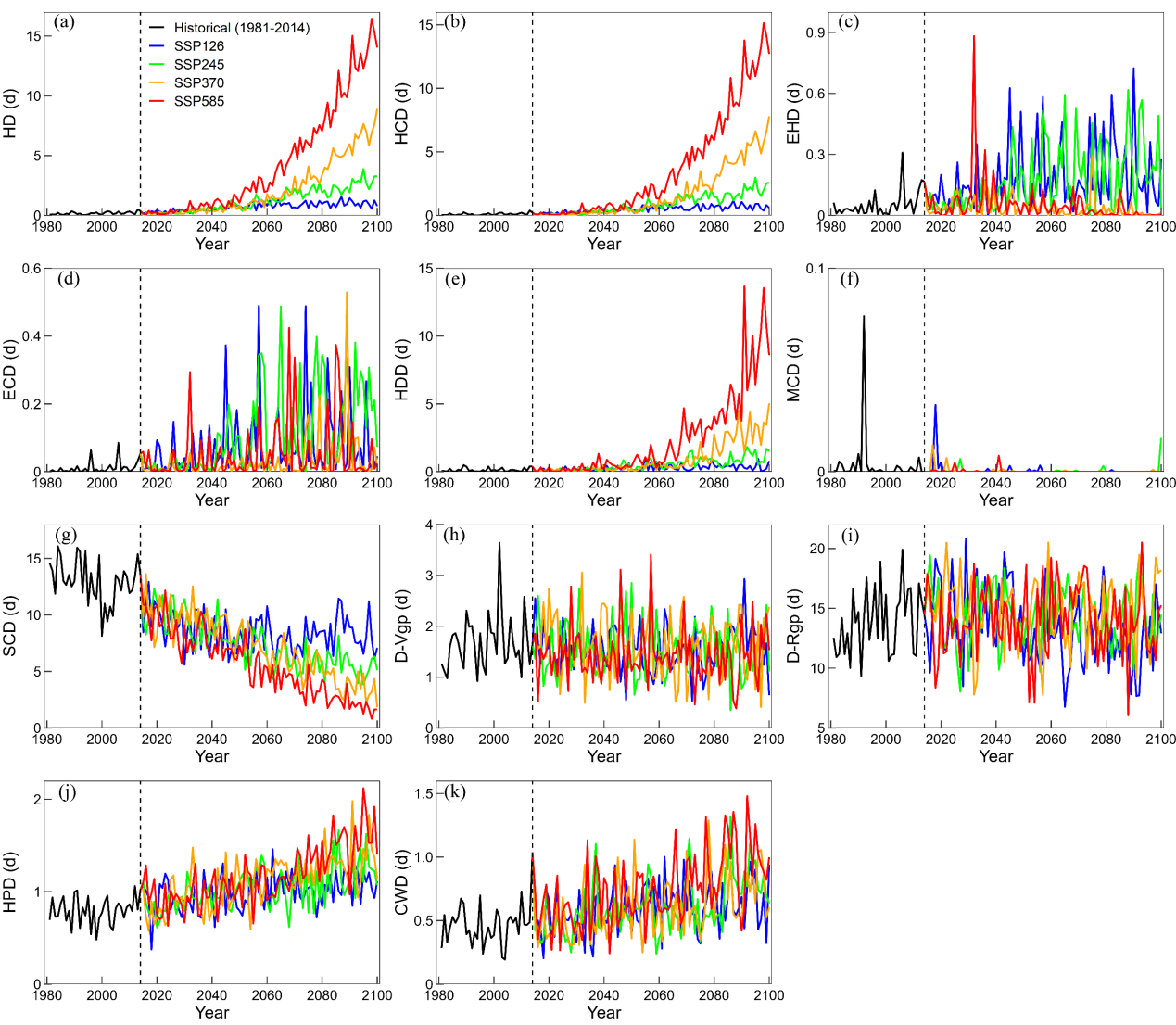


**Figure S10.** The trends of 11 extreme climate indices (ECIs) in Zone Ⅴ during the historical period and 4 future climate scenarios of late-rice.


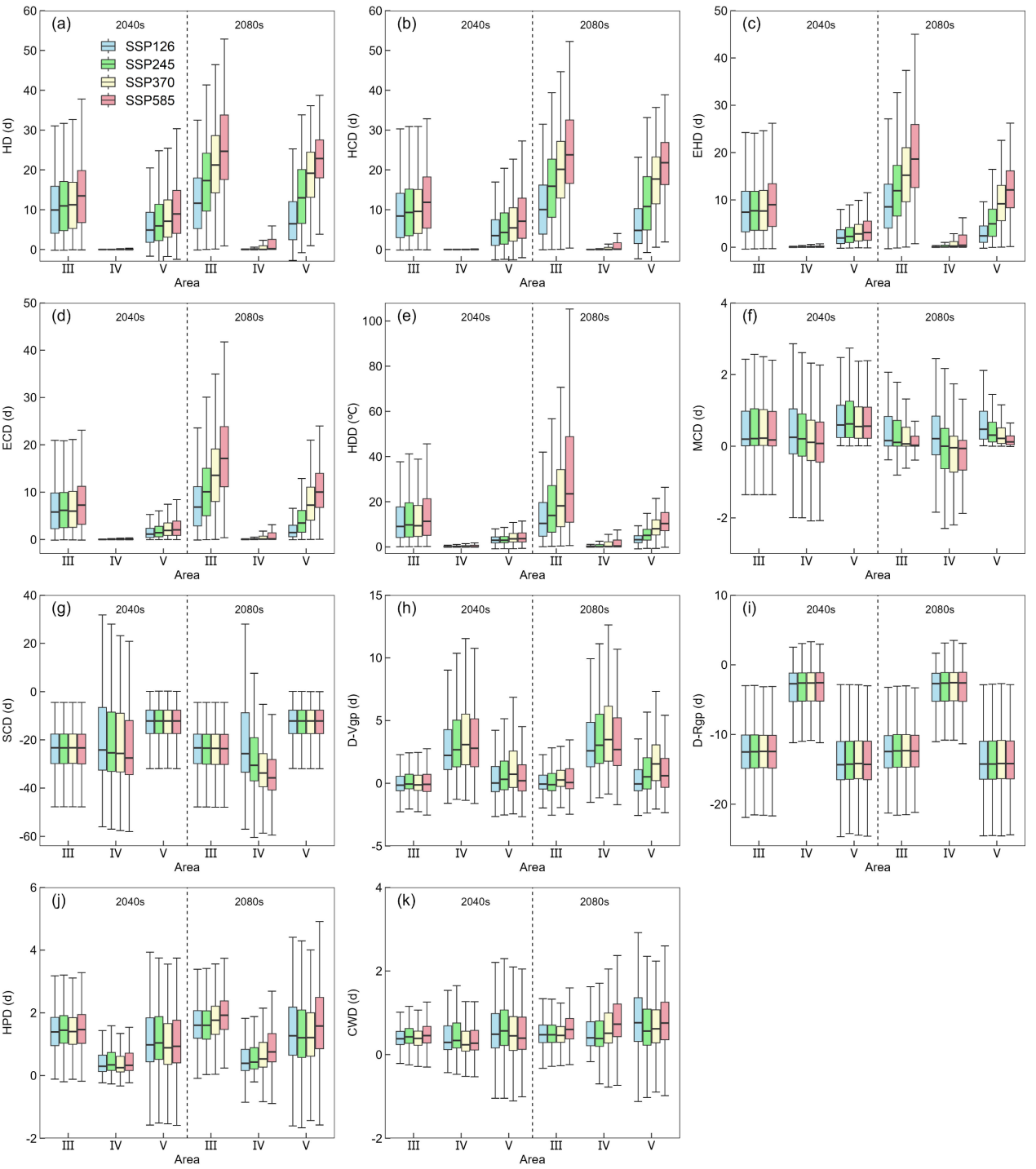


**Figure S11.** The changes in the 11 extreme climate indices (ECIs) under the 4 future (2040s and 2080s) climate scenarios compared to the historical periods of late-rice.


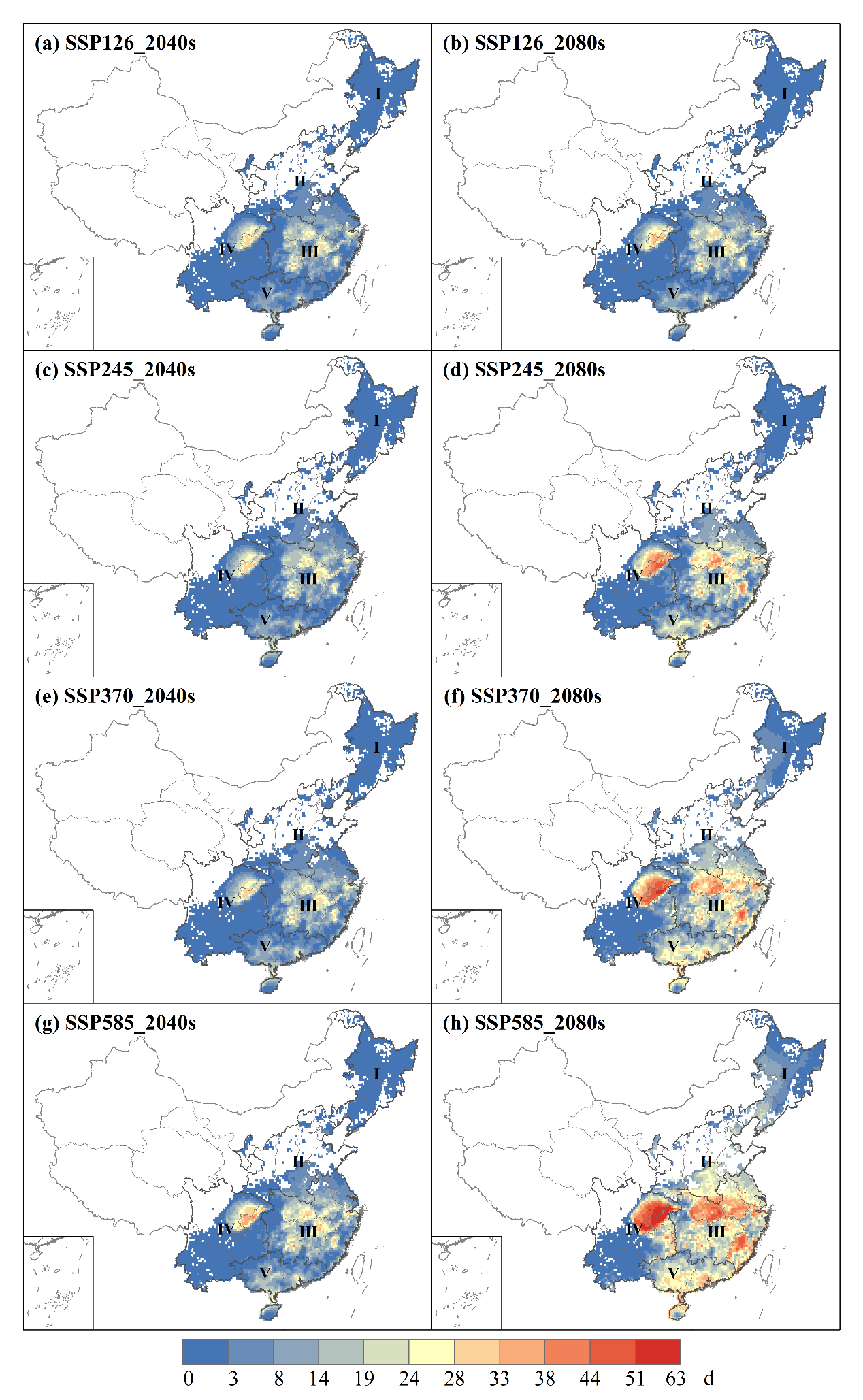


**Figure S12.** The spatial distribution of HCD at 2040s and 2080s under 4 future climate scenarios of single-rice and early-rice.


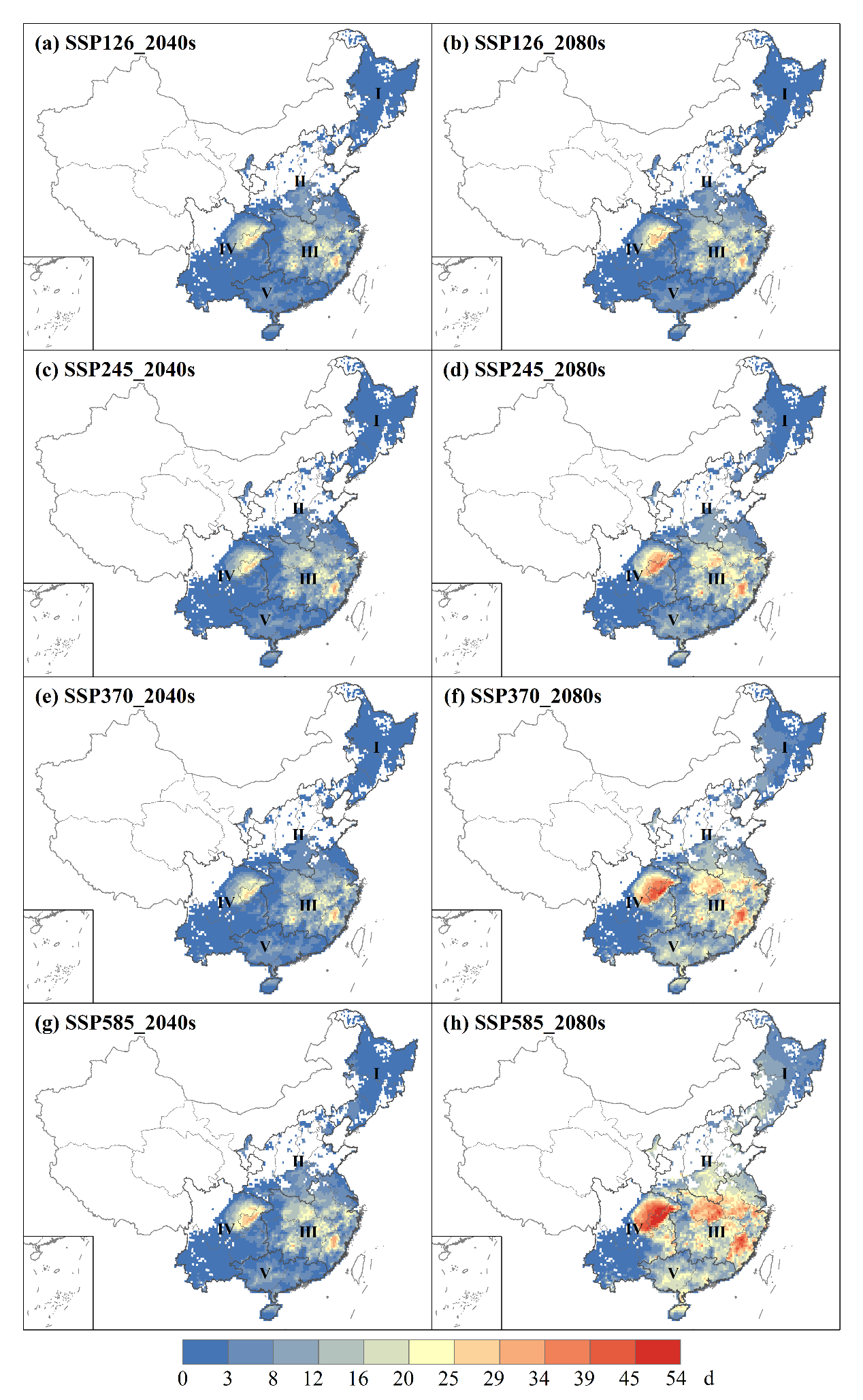


**Figure S13.** The spatial distribution of EHD at 2040s and 2080s under 4 future climate scenarios of single-rice and early-rice.


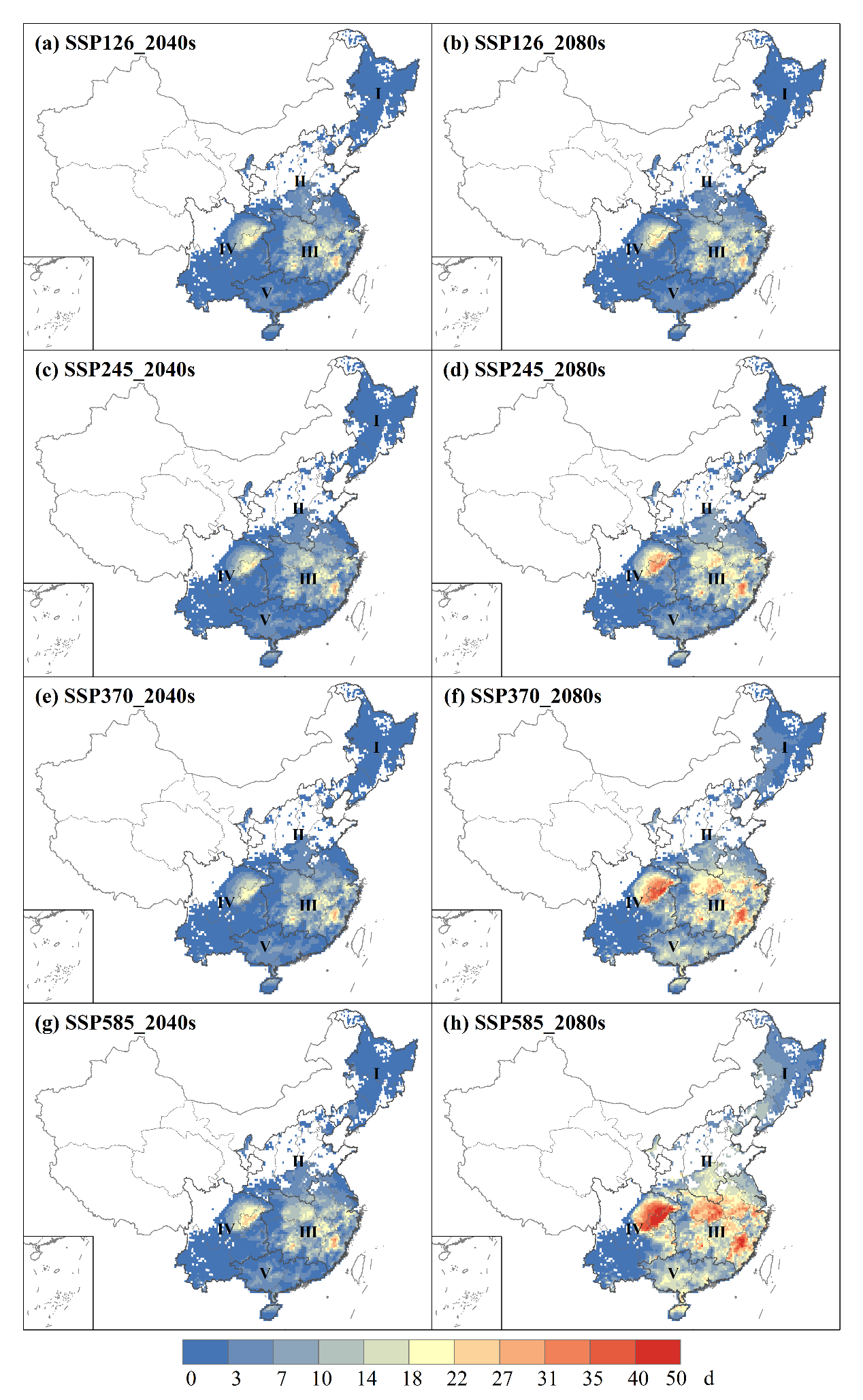


**Figure S14.** The spatial distribution of ECD at 2040s and 2080s under 4 future climate scenarios of single-rice and early-rice.


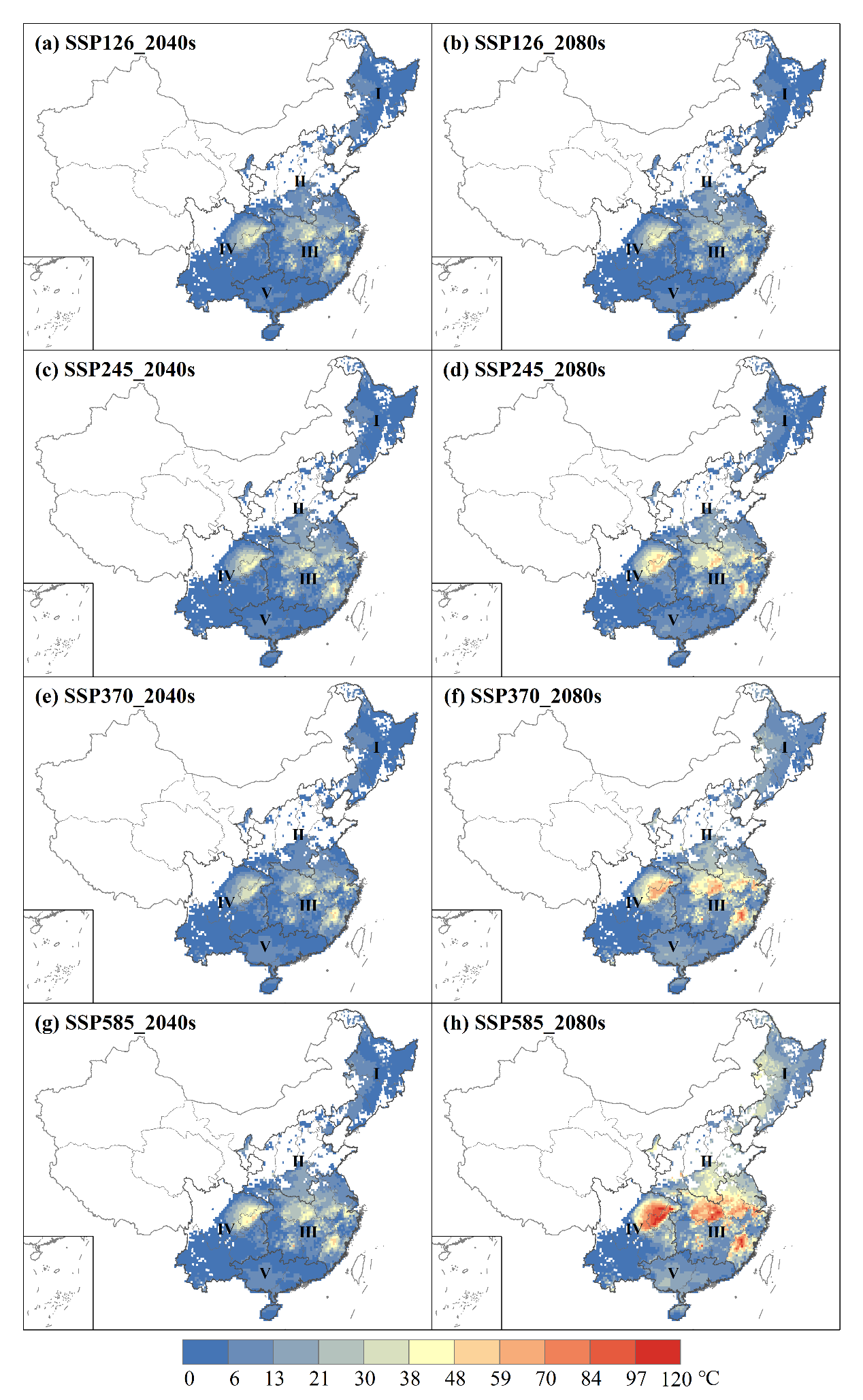


**Figure S15.** The spatial distribution of HDD at 2040s and 2080s under 4 future climate scenarios of single-rice and early-rice.


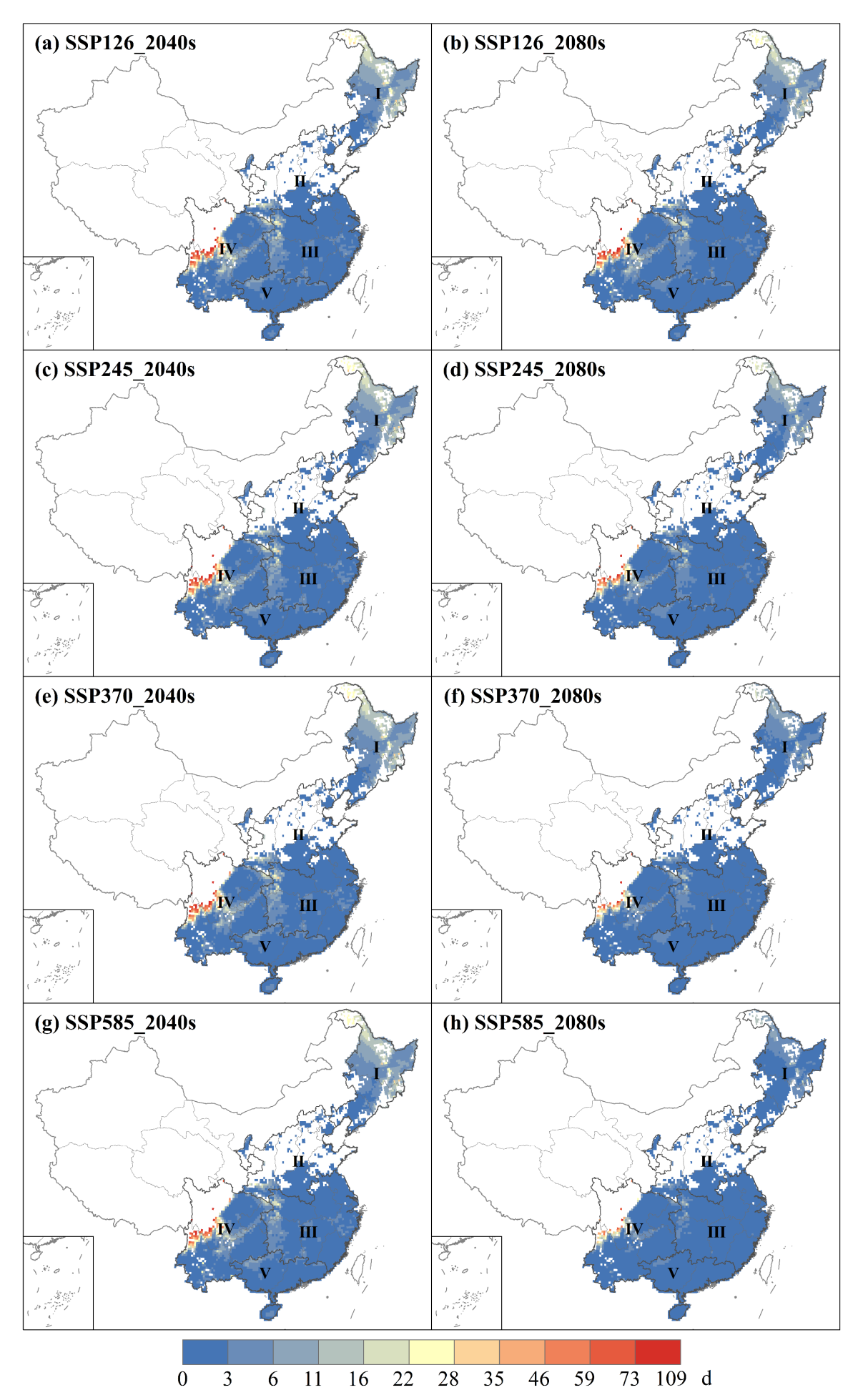


**Figure S16.** The spatial distribution of MCD at 2040s and 2080s under 4 future climate scenarios of single-rice and early-rice.


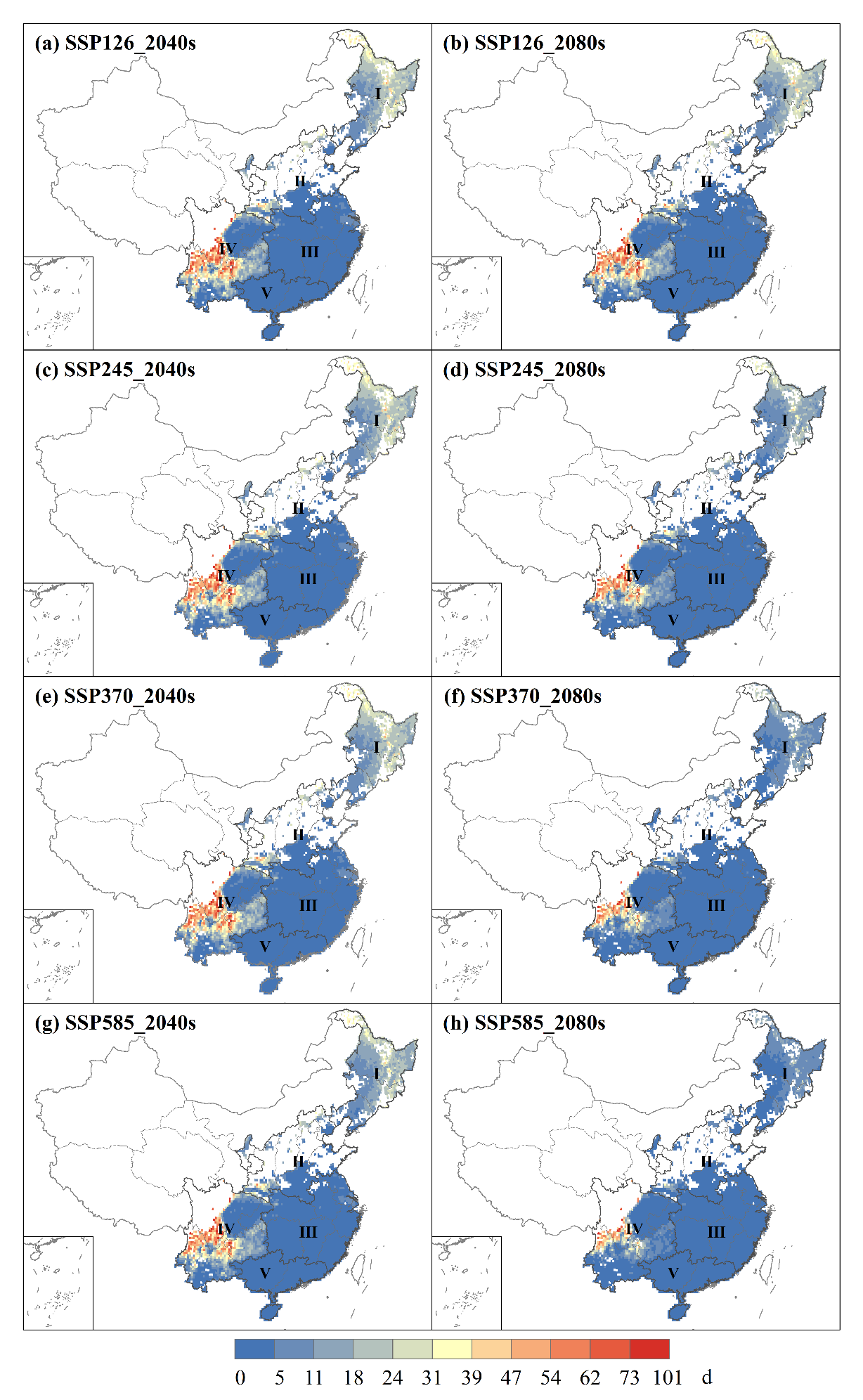


**Figure S17.** The spatial distribution of SCD at 2040s and 2080s under 4 future climate scenarios of single-rice and early-rice.


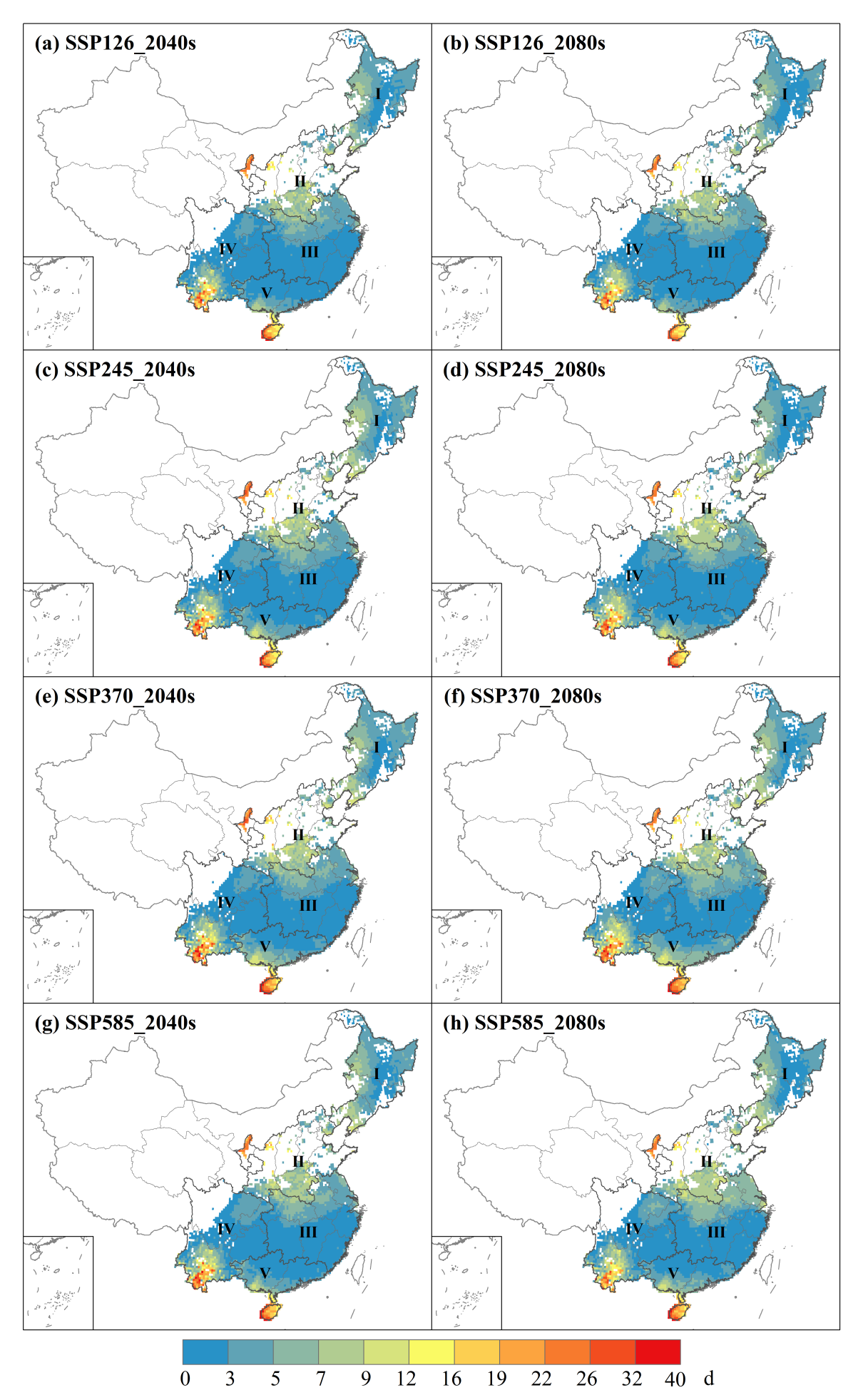


**Figure S18.** The spatial distribution of D-Vgp at 2040s and 2080s under 4 future climate scenarios of single-rice and early-rice.


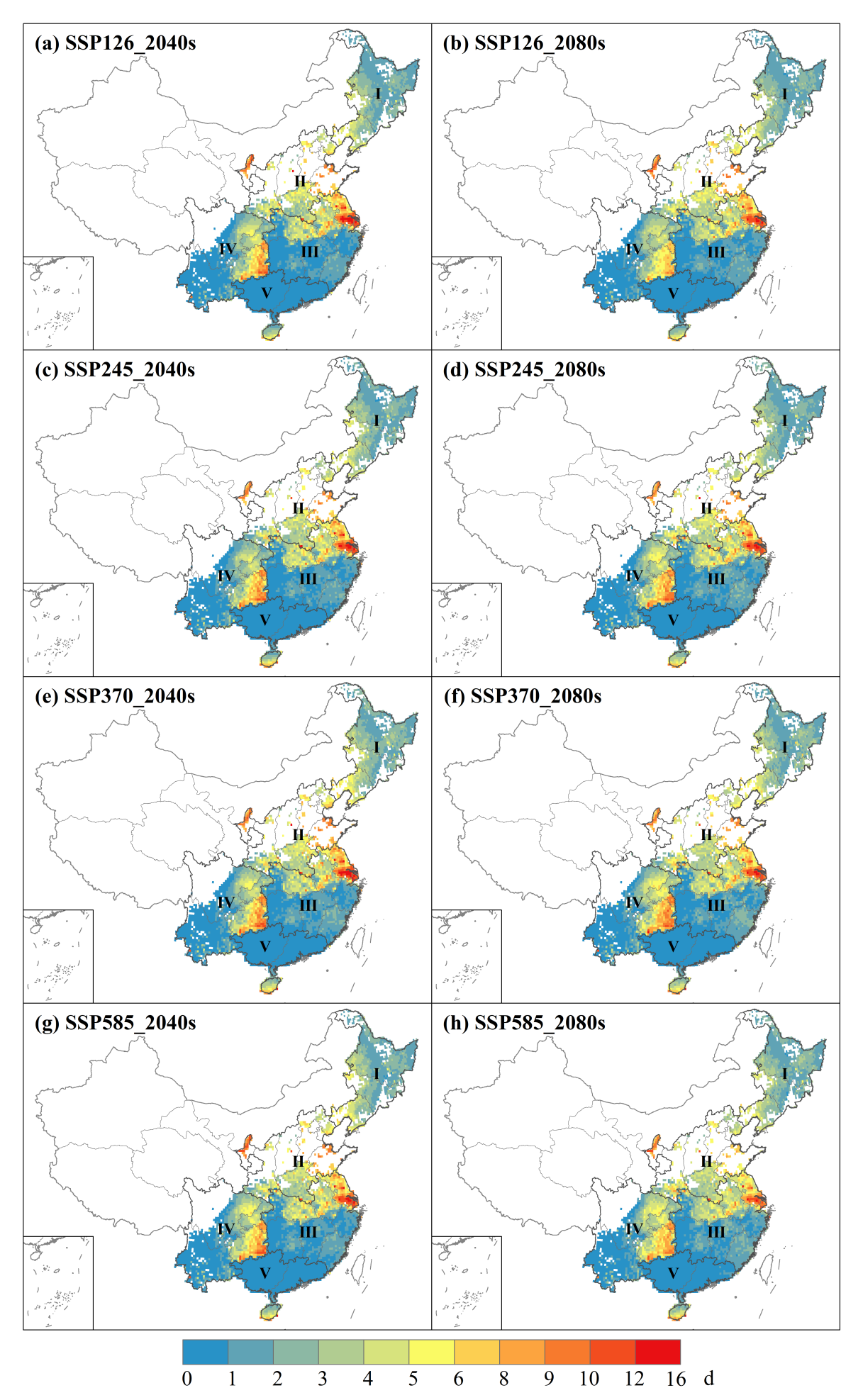


**Figure S19.** The spatial distribution of D-Rgp at 2040s and 2080s under 4 future climate scenarios of single-rice and early-rice.


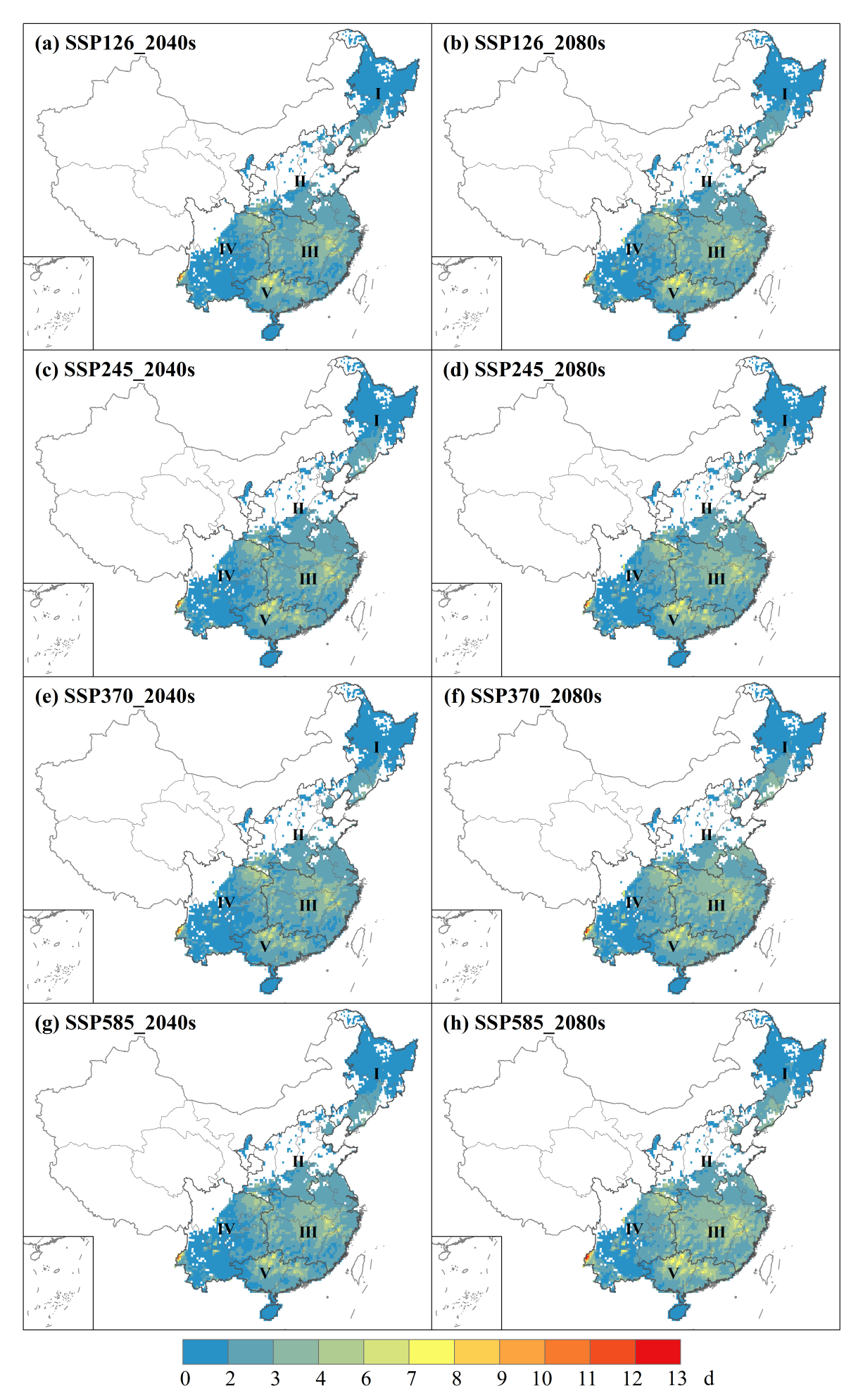


**Figure S20.** The spatial distribution of HPD at 2040s and 2080s under 4 future climate scenarios of single-rice and early-rice.


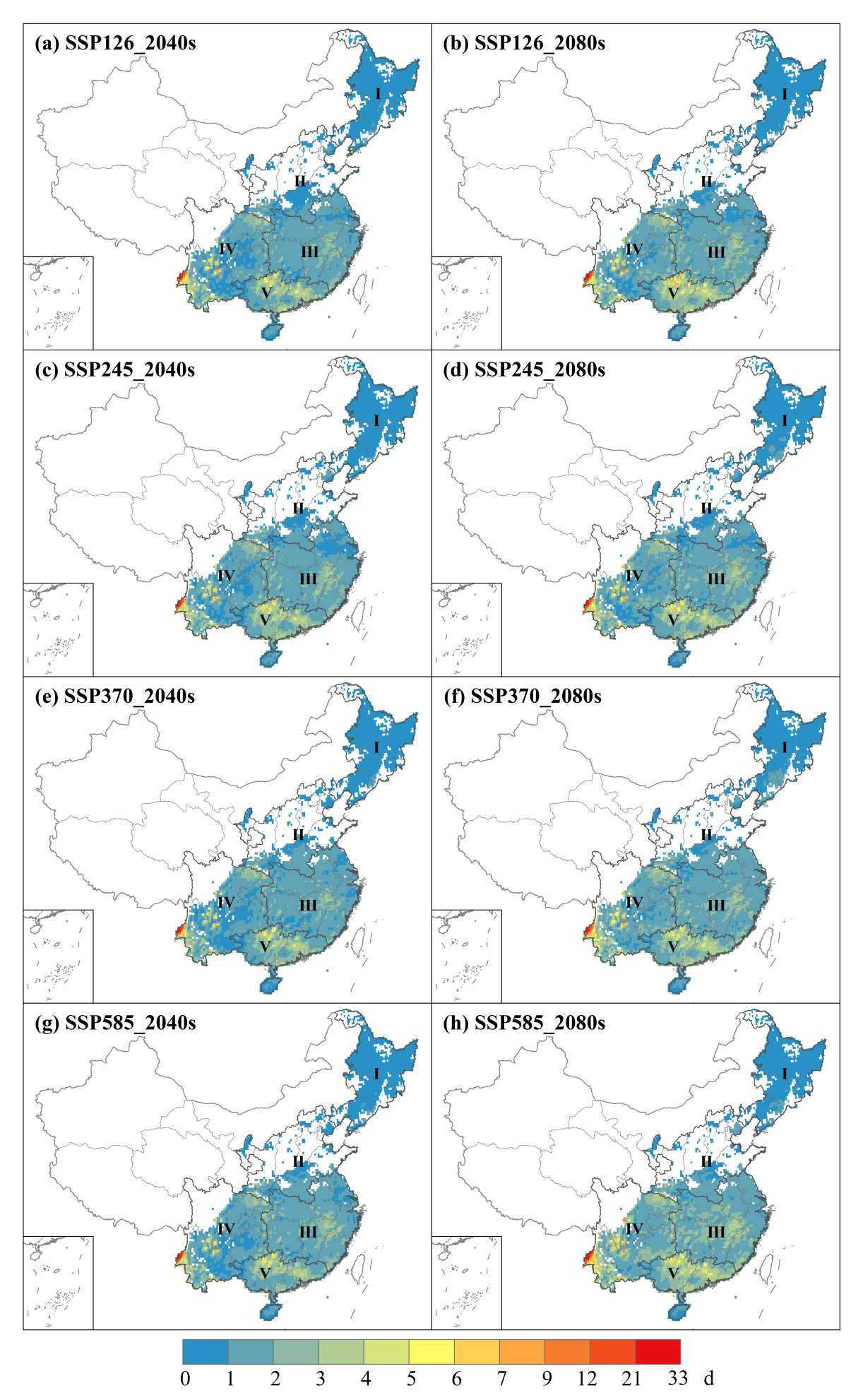


**Figure S21.** The spatial distribution of CWD at 2040s and 2080s under 4 future climate scenarios of single-rice and early-rice.


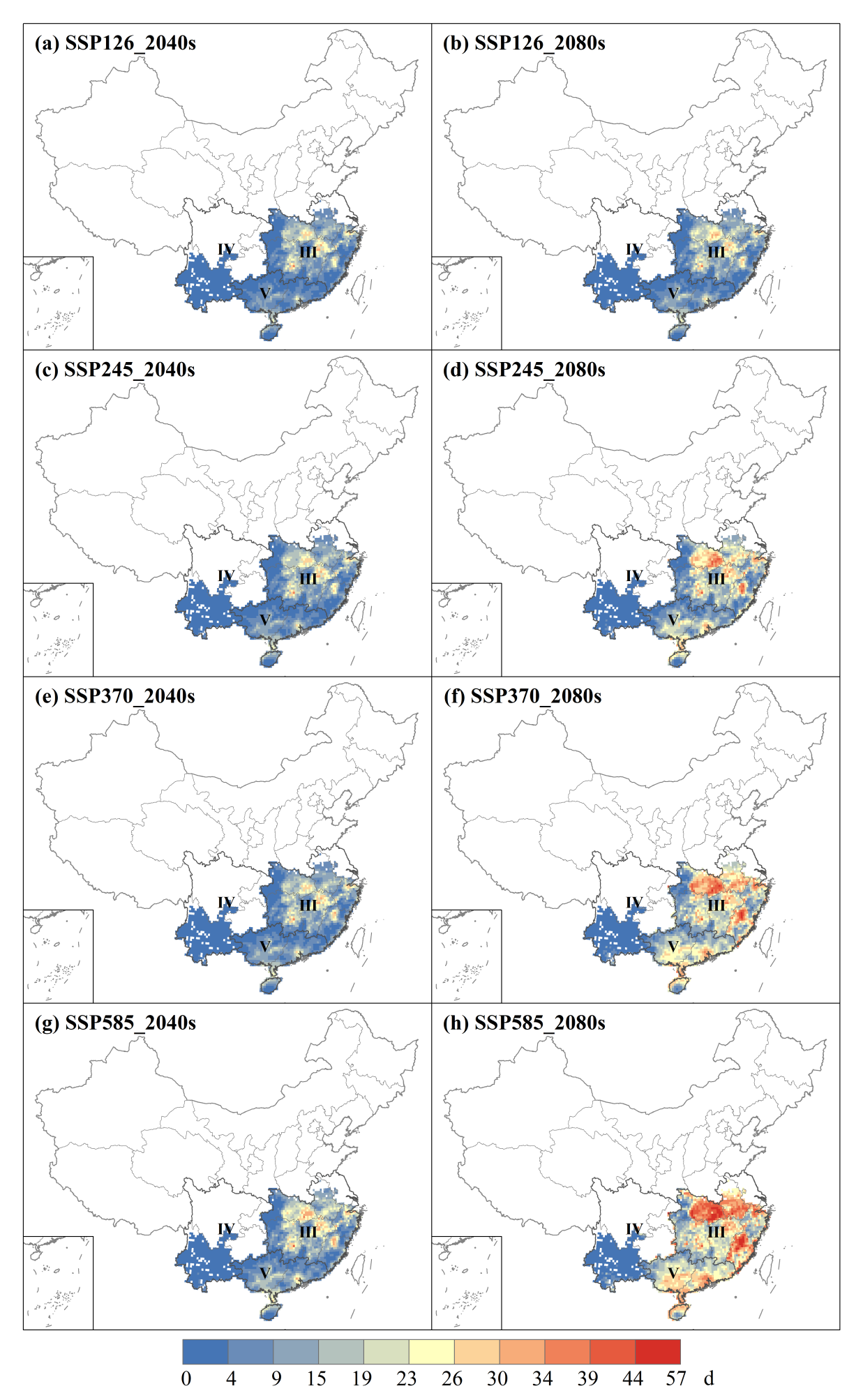
 **Figure S22.** The spatial distribution of HCD at 2040s and 2080s under 4 future climate scenarios of late-rice.


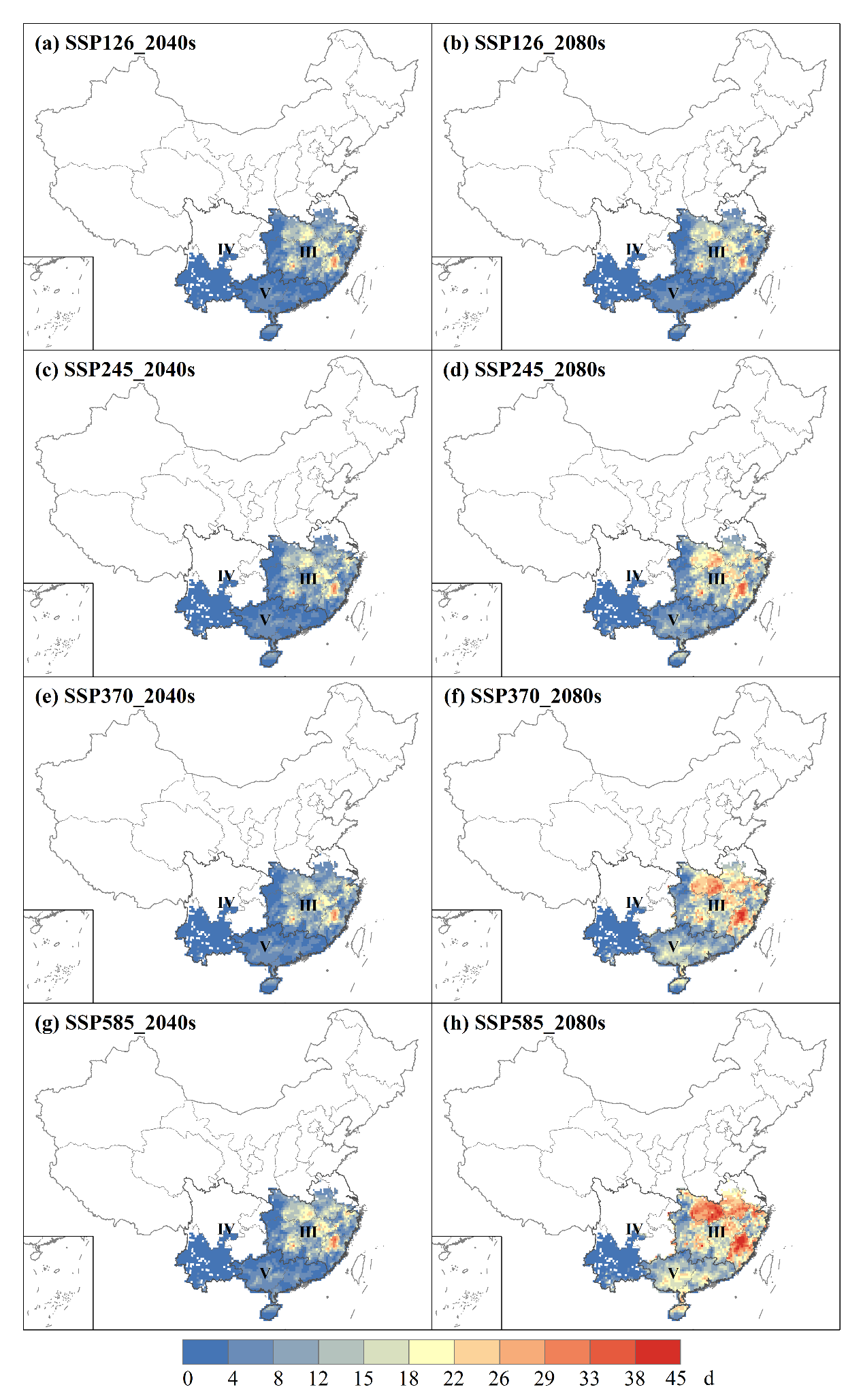


**Figure S23.** The spatial distribution of EHD at 2040s and 2080s under 4 future climate scenarios of late-rice.


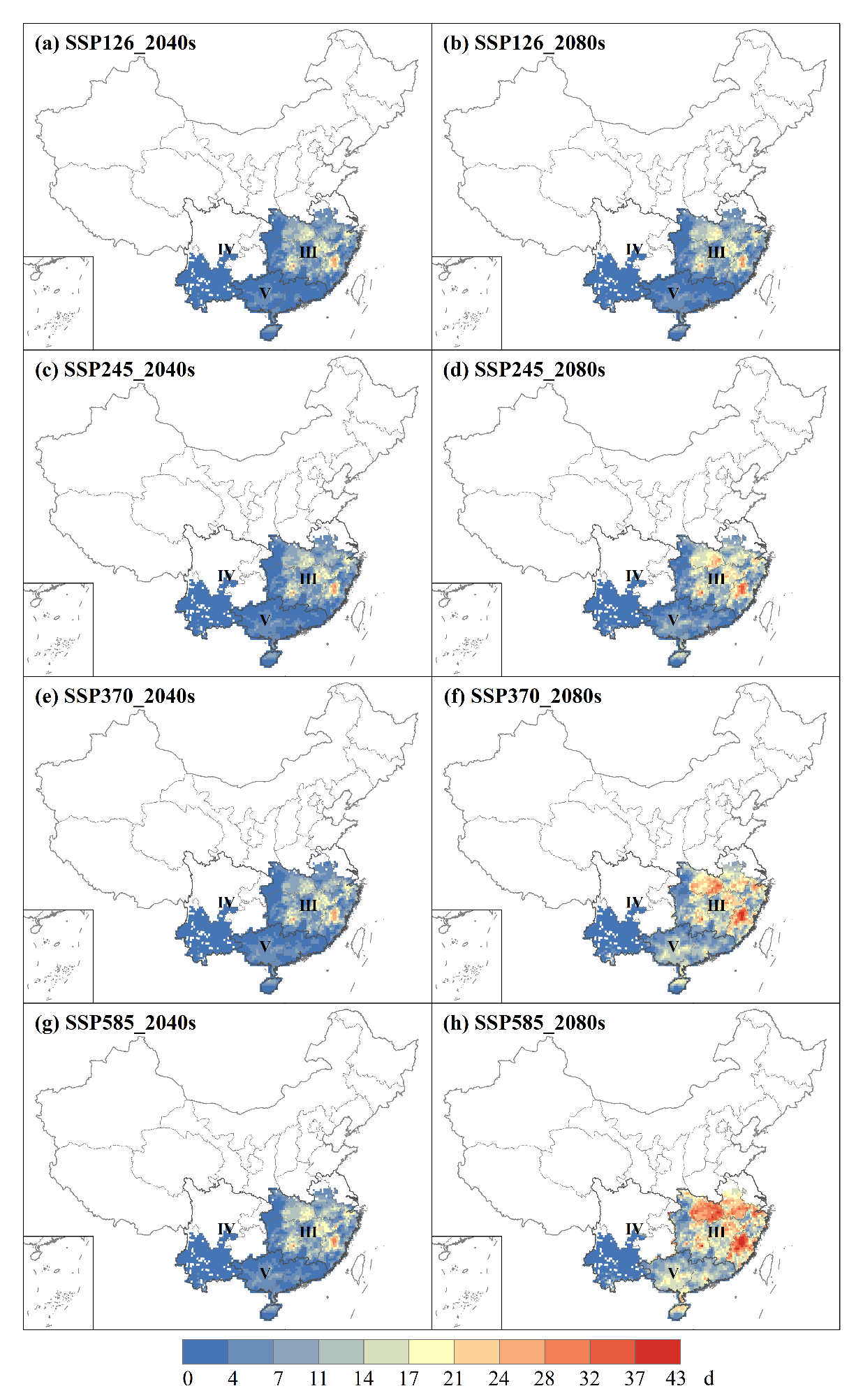


**Figure S24.** The spatial distribution of ECD at 2040s and 2080s under 4 future climate scenarios of late-rice.


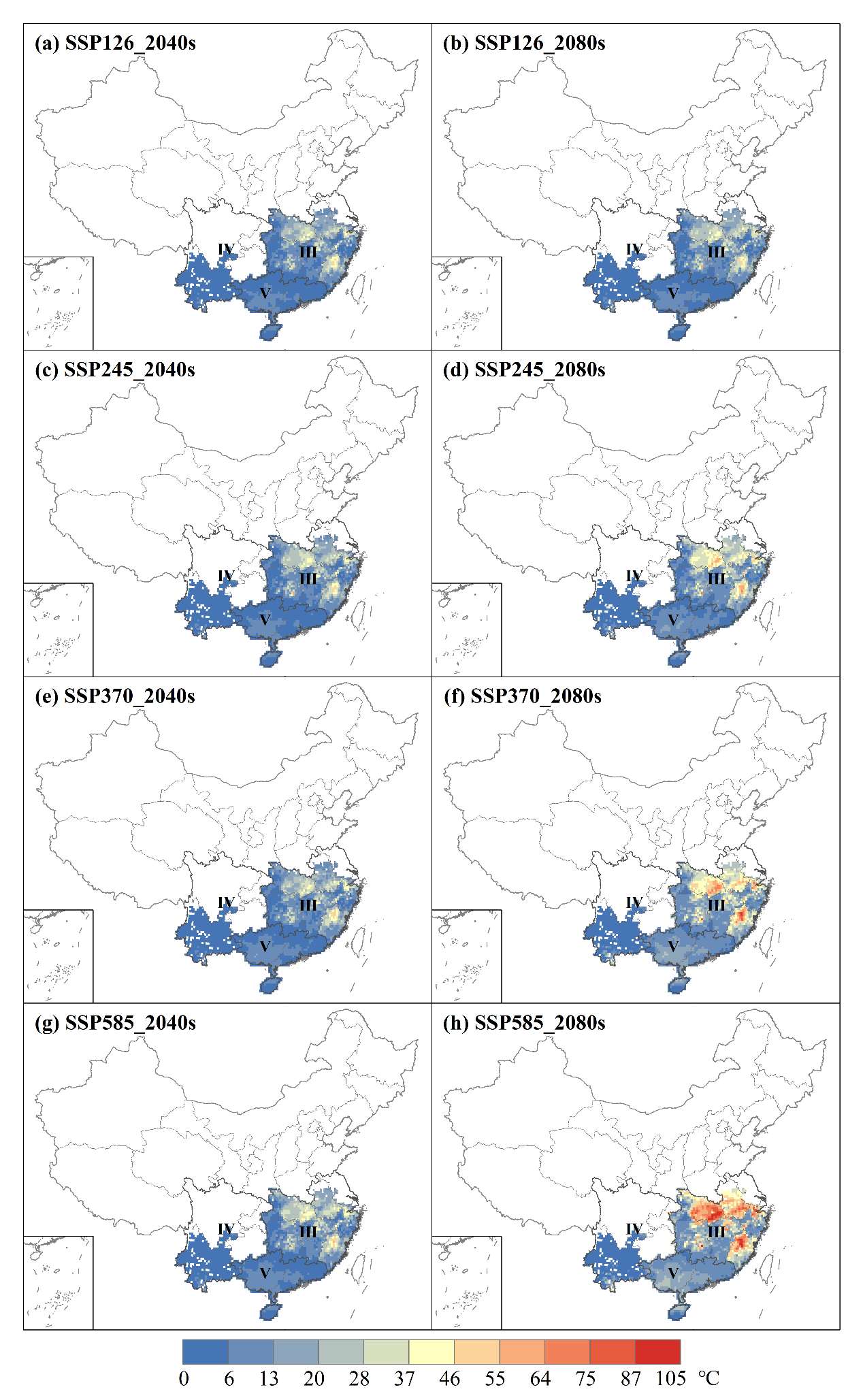


**Figure S25.** The spatial distribution of HDD at 2040s and 2080s under 4 future climate scenarios of late-rice.


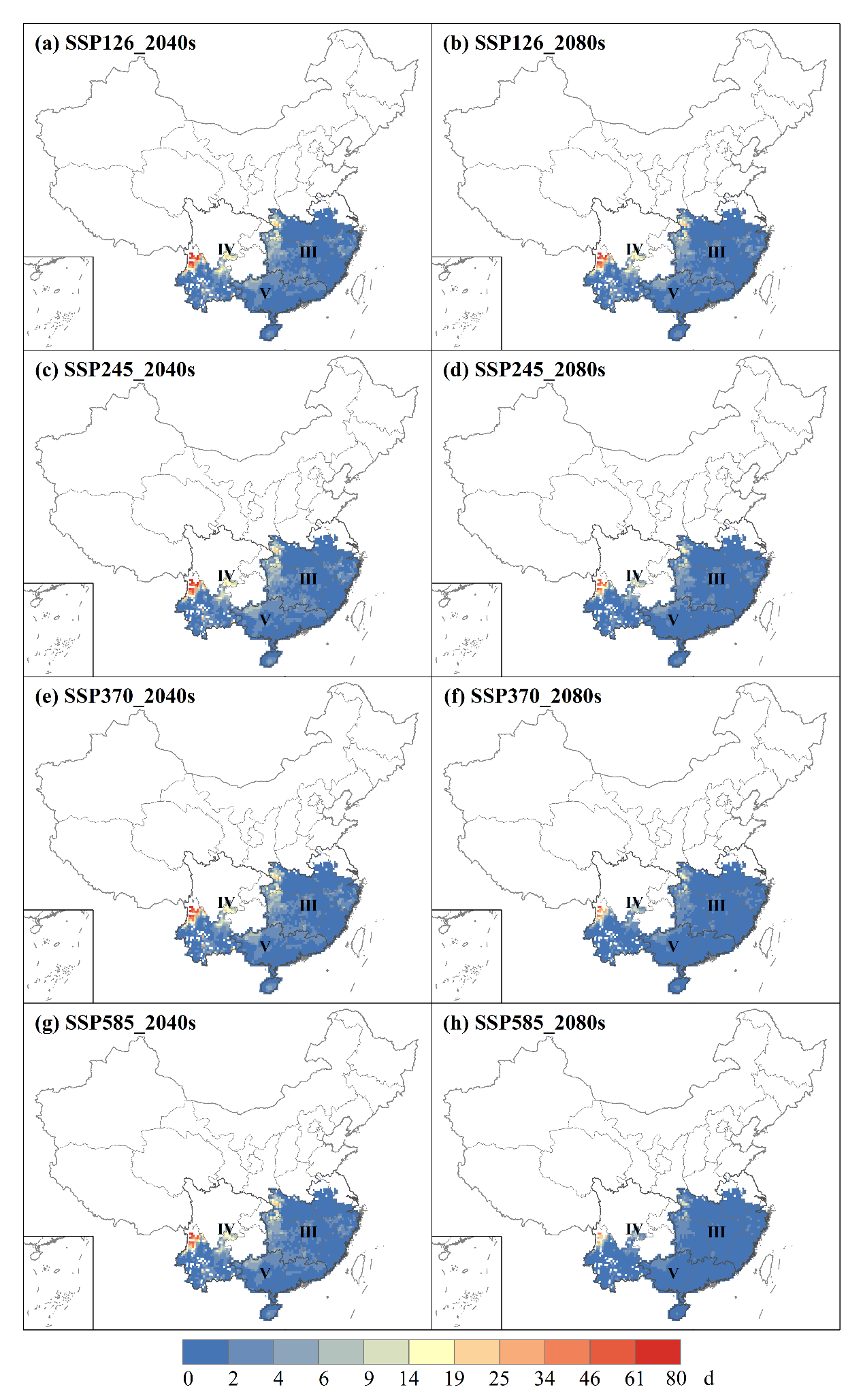


**Figure S26.** The spatial distribution of MCD at 2040s and 2080s under 4 future climate scenarios of late-rice.


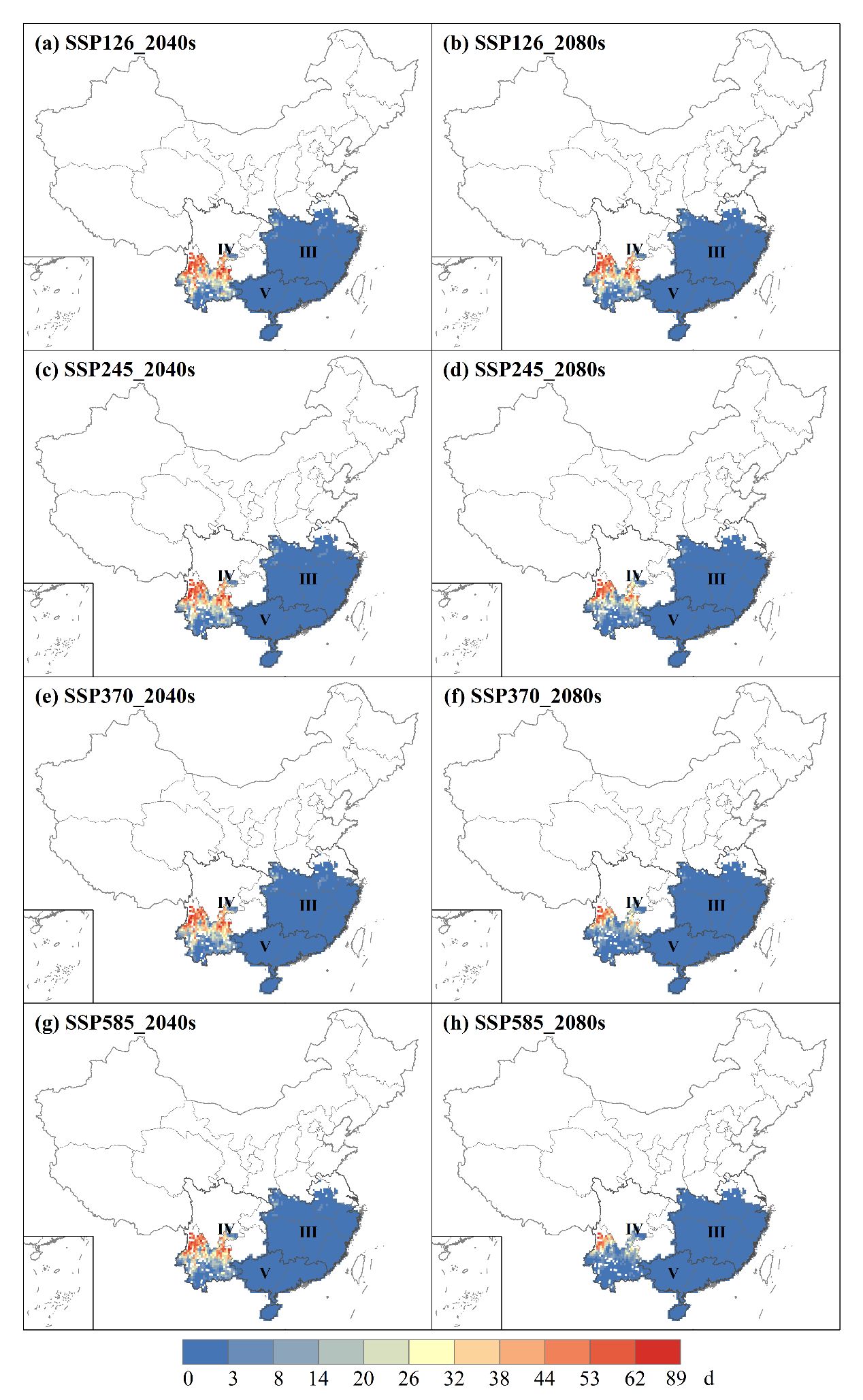


**Figure S27.** The spatial distribution of SCD at 2040s and 2080s under 4 future climate scenarios of late-rice.


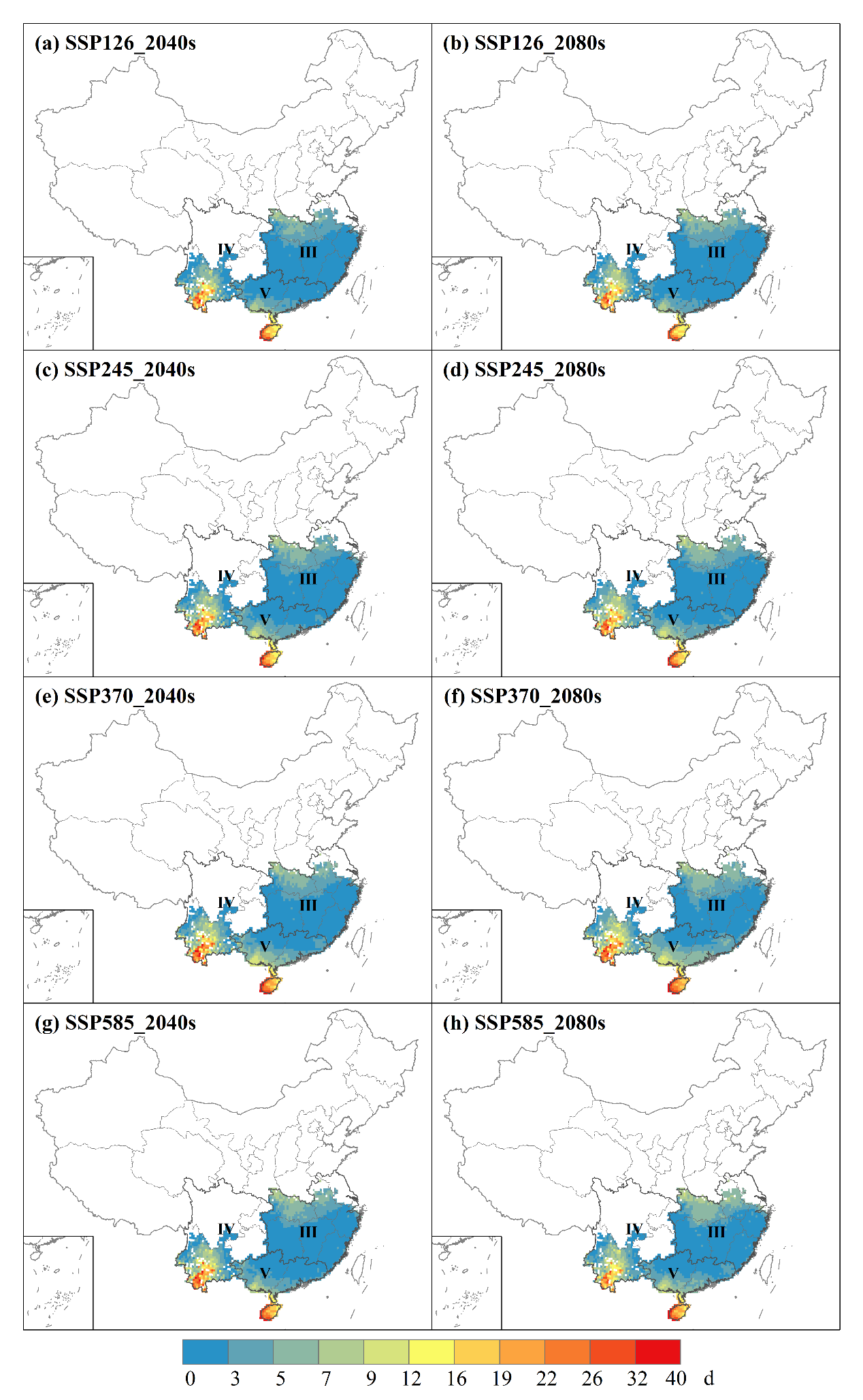


**Figure S28.** The spatial distribution of D-Vgp at 2040s and 2080s under 4 future climate scenarios of late-rice.


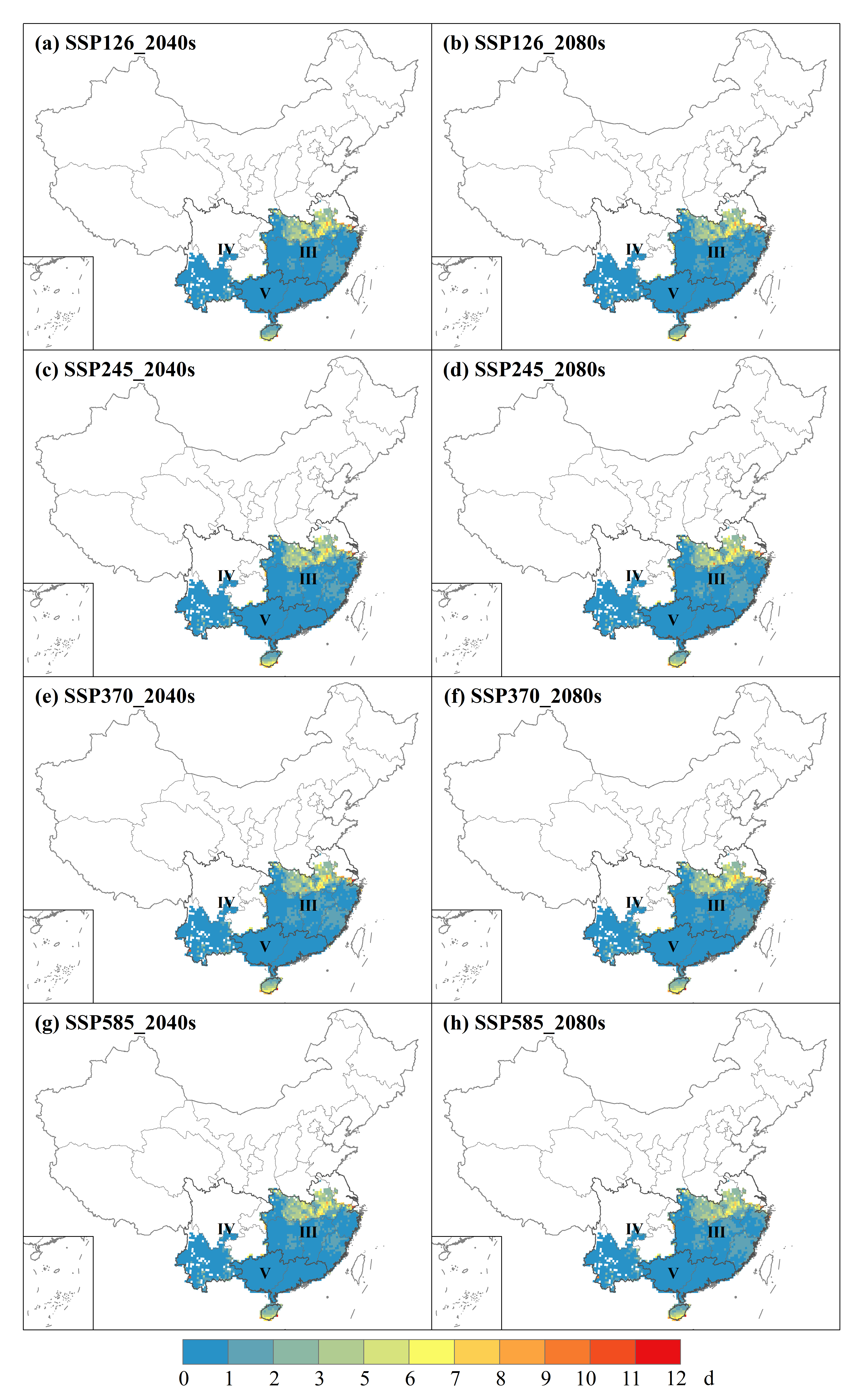


**Figure S29.** The spatial distribution of D-Rgp at 2040s and 2080s under 4 future climate scenarios of late-rice.


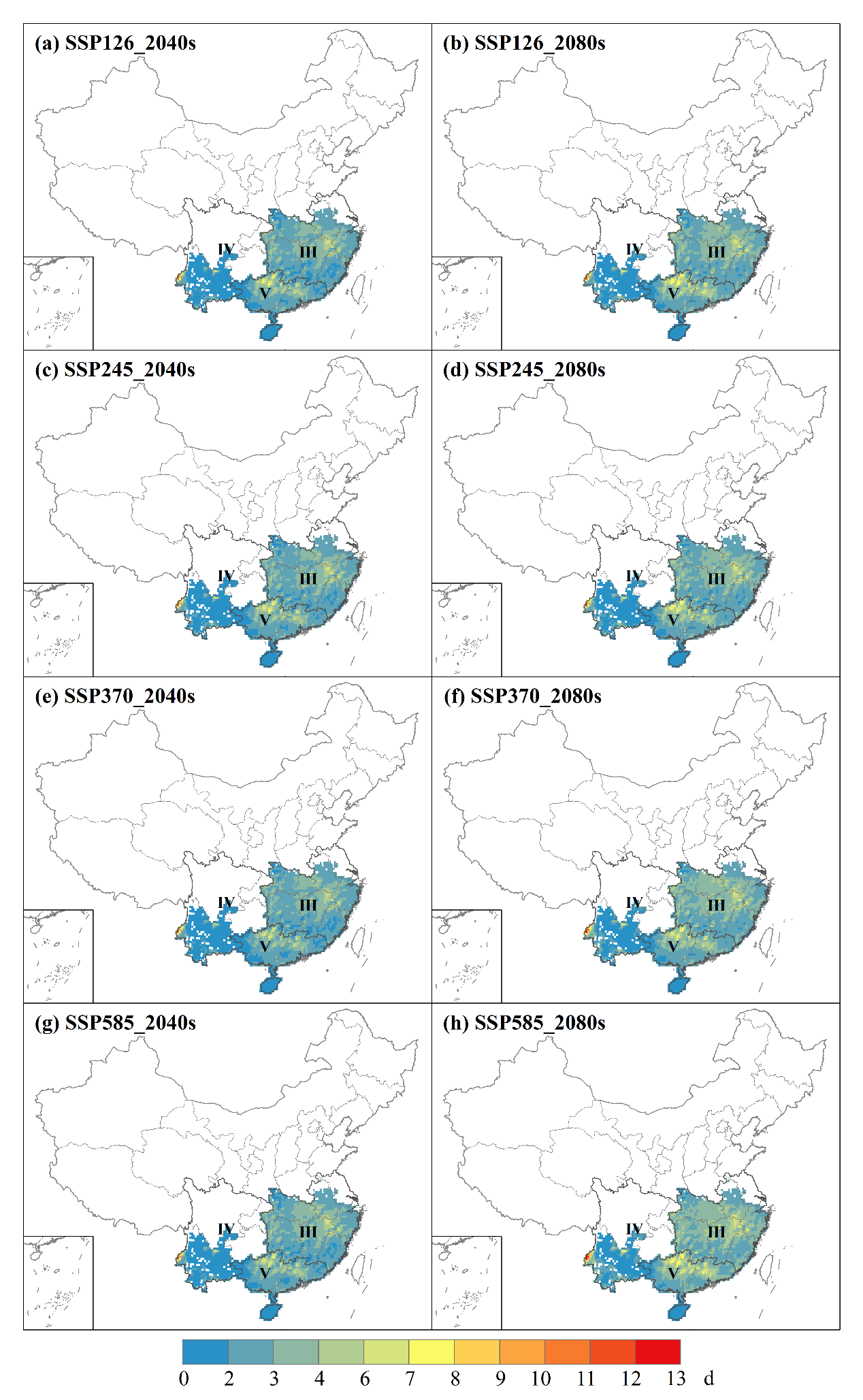


**Figure S30.** The spatial distribution of HPD at 2040s and 2080s under 4 future climate scenarios of late-rice.


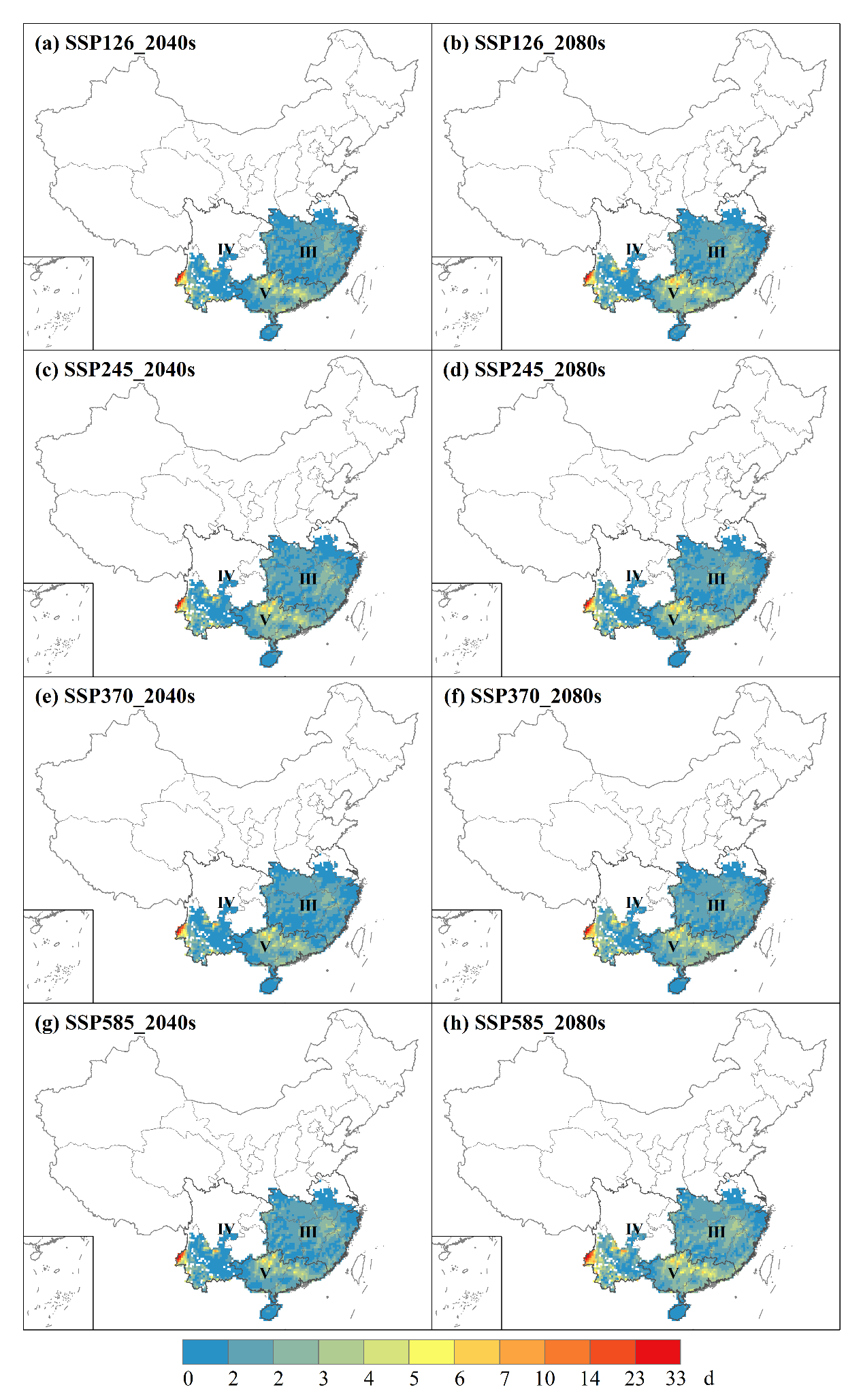


**Figure S31.** The spatial distribution of CWD at 2040s and 2080s under 4 future climate scenarios of late-rice.
